# Supplementary material for: Discovery and characterization of genes conferring natural resistance to the antituberculosis antibiotic capreomycin
Source: Commun Biol. 2023 Dec 19;6:1282. doi: 10.1038/s42003-023-05681-6 (PMC10730852; doi:10.1038/s42003-023-05681-6)
Supplement: Supplementary file 2 — Supplementary Information [file 42003_2023_5681_MOESM2_ESM.pdf]

Supplementary Information

Discovery and Characterization of Genes Conferring Natural Resistance to the Antituberculosis Antibiotic Capreomycin

Shu-Ing Toh, Johan Elaine Keisha, Yung-Lin Wang, Yi-Chi Pan, Yu-Heng Jhu, Po-Yun Hsiao, Wen-Ting Liao, Po-Yuan Chen, Tai-Ming Ko, and Chin-Yuan Chang*

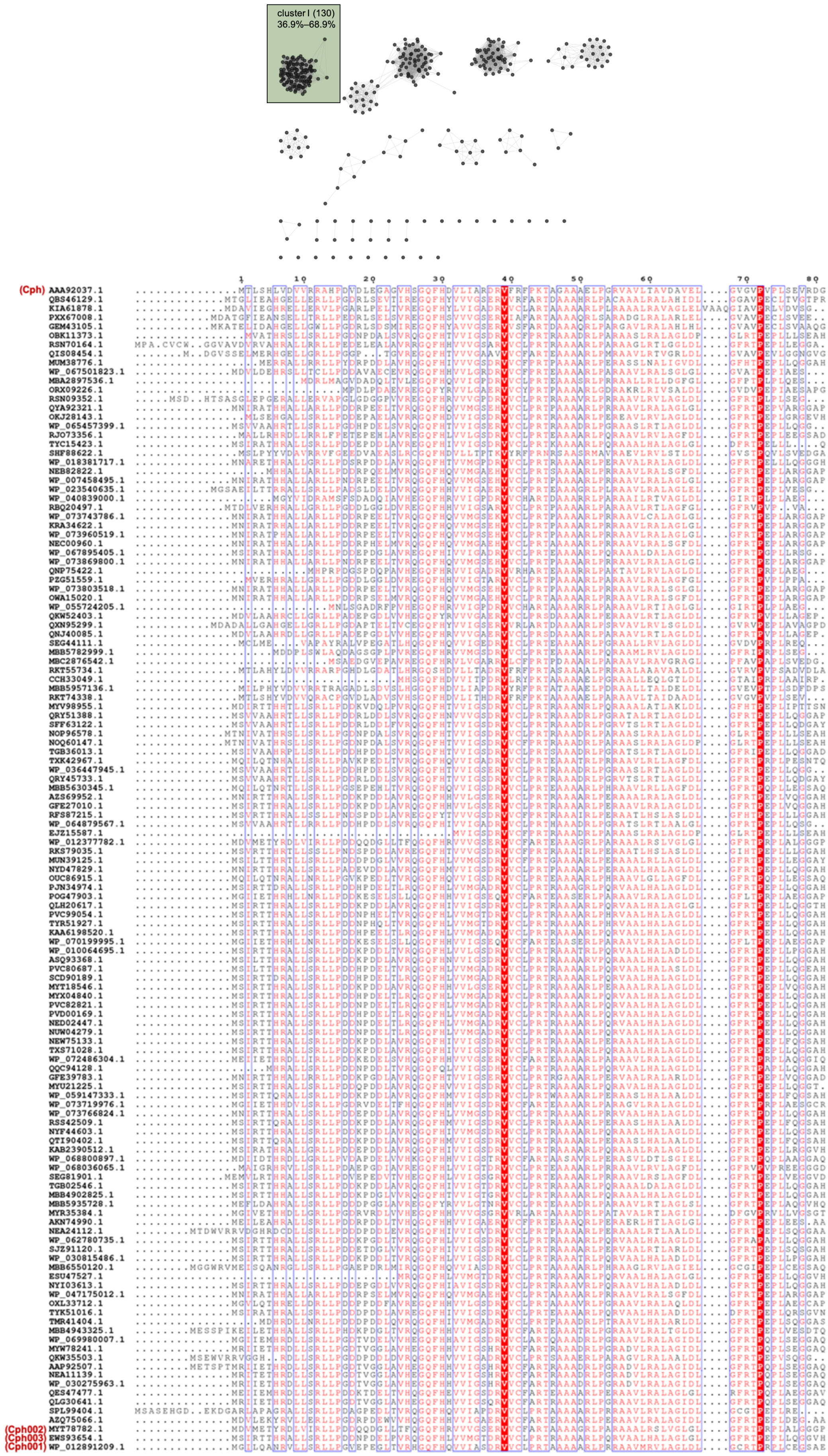

cluster I (130)
36.9%–68.9%
(Cph) AAA92037.1
(Cph002) MYT78782.1
(Cph003) EWS93654.1
(Cph001) WP_012891209.1

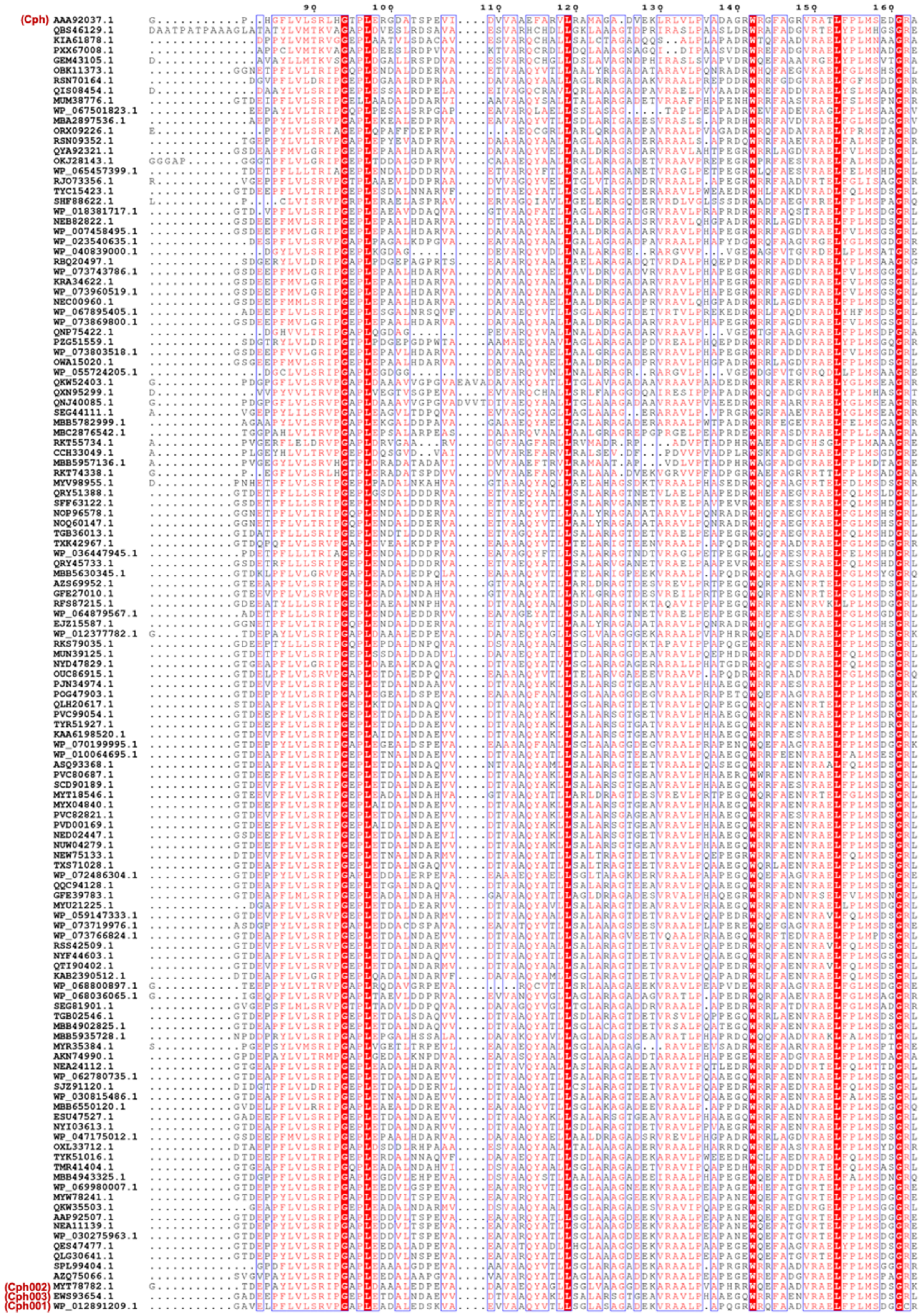
90
100
110
120
130
140
150
160
(Cph) AAA92037.1
(Cph002) MYT78782.1
(Cph003) EWS93654.1
(Cph001) WP_012891209.1

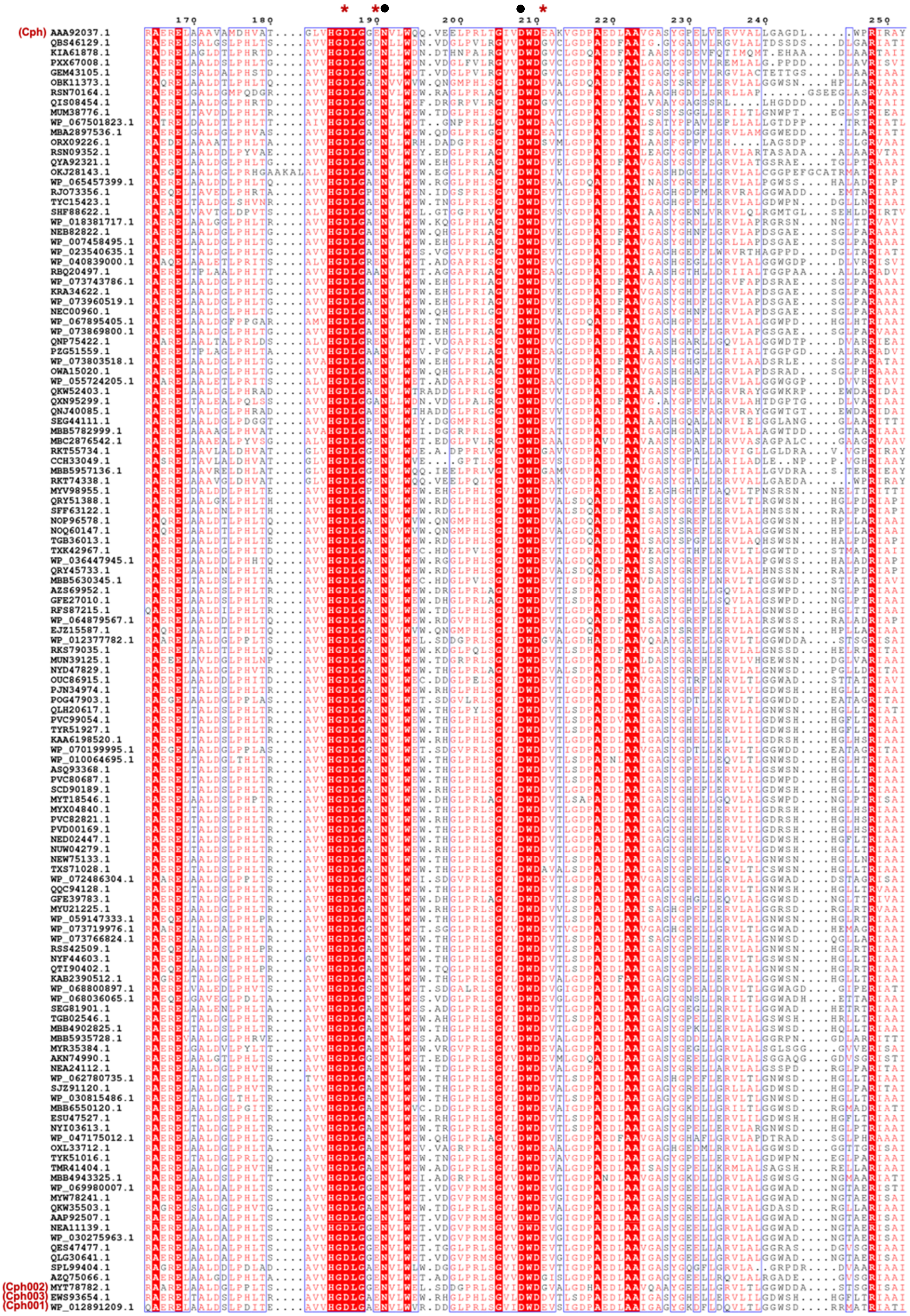
(Cph)
(Cph002)
(Cph003)
(Cph001)

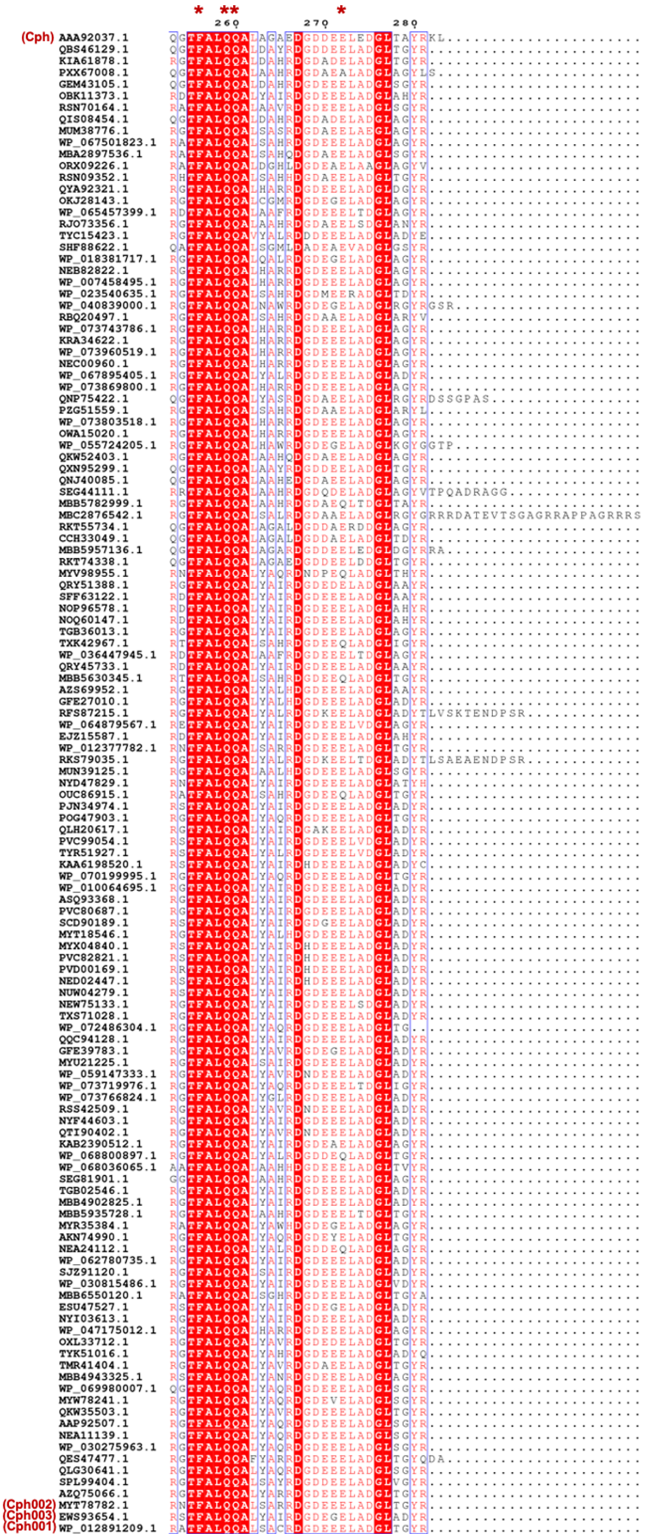

Supplementary Fig. 1 Sequence alignment of Cph with proteins from cluster I.
Aligned residues are colored on the bases of the level of conservation (red background shows identity, red character for similarity, and blue frame for similarity across group). The residues involved in CMN binding in Cph are marked with an red asterisk (*) above the sequence alignment. The putative Mg^{2+}-binding residues are marked with a black dot (●) above the sequence alignment.

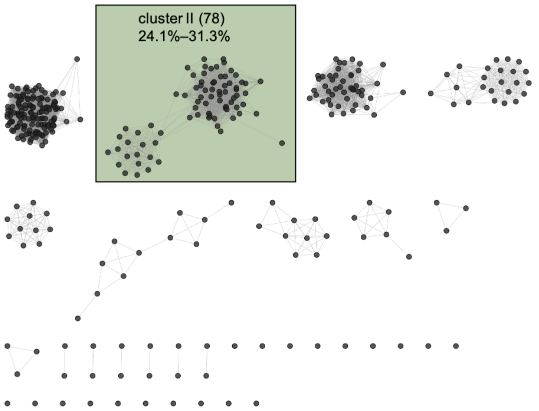
cluster II (78)
24.1%–31.3%

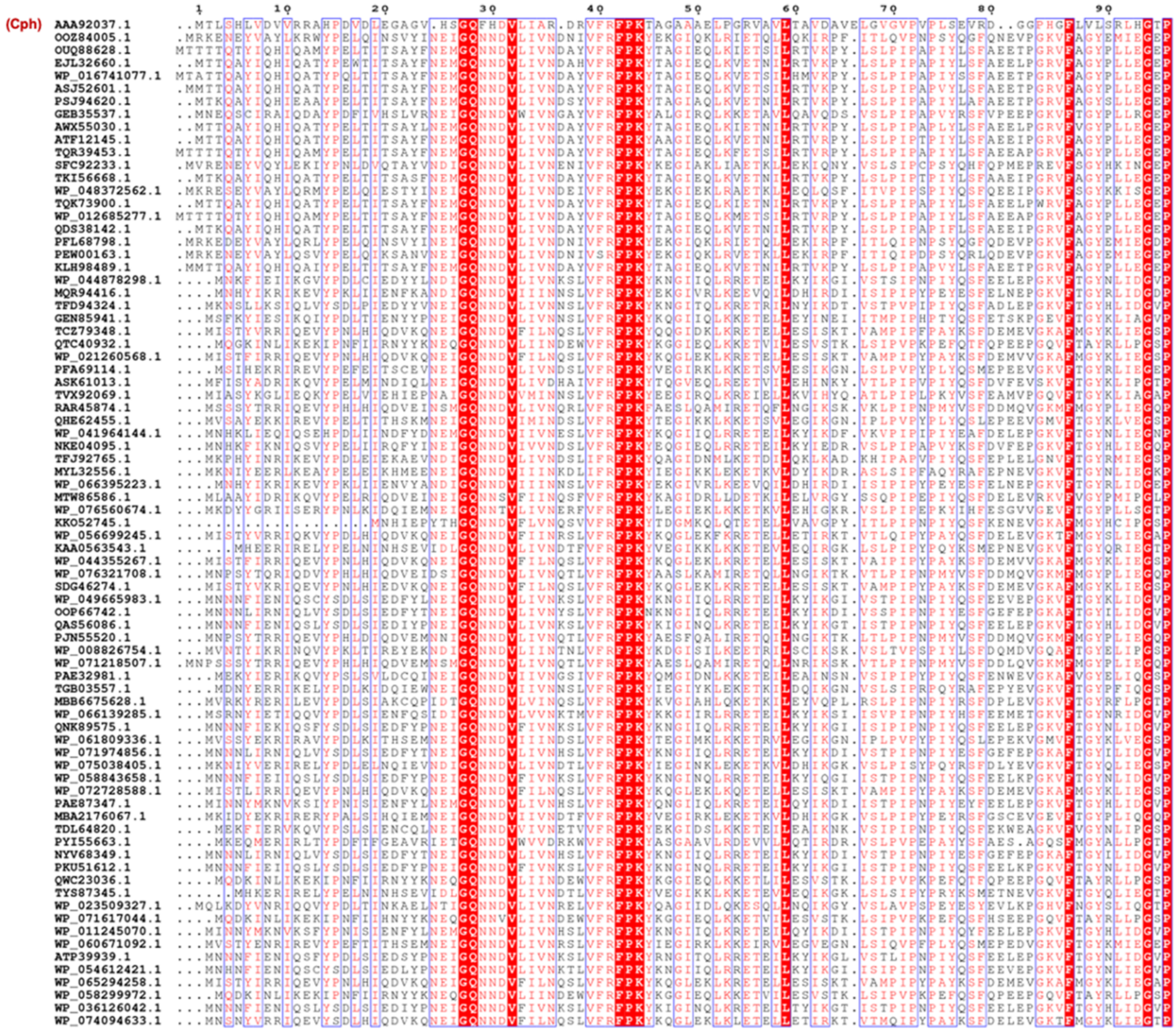
1 10 20 30 40 50 60 70 80 90
(Cph)
AAA92037.1
OOZ84005.1
OUQ88628.1
EJL32660.1
WP_016741077.1
ASJ52601.1
PSJ94620.1
GEB35537.1
AWX55030.1
ATF12145.1
TQR39453.1
SFC92233.1
TKI56668.1
WP_048372562.1
TQK73900.1
WP_012685277.1
QDS38142.1
PFL68798.1
PEW00163.1
KLH98489.1
WP_044878298.1
MQR94416.1
TFD94324.1
GEN85941.1
TCZ79348.1
QTC40932.1
WP_021260568.1
PFA69114.1
ASK61013.1
TVX92069.1
RAR45874.1
QHE62455.1
WP_041964144.1
NKE04095.1
TFJ92765.1
MYL32556.1
WP_066395223.1
MTW86586.1
WP_076560674.1
KKO52745.1
WP_056699245.1
KAA0563543.1
WP_044355267.1
WP_076321708.1
SDG46274.1
WP_049665983.1
OOP66742.1
QAS56086.1
PJN55520.1
WP_008826754.1
WP_071218507.1
PAE32981.1
TGB03557.1
MBB6675628.1
WP_066139285.1
QNK89575.1
WP_061809336.1
WP_071974856.1
WP_075038405.1
WP_058843658.1
WP_072728588.1
PAE87347.1
MBA2176067.1
TDL64820.1
PYI55663.1
NYV68349.1
PKU51612.1
QWC23036.1
TYS87345.1
WP_023509327.1
WP_071617044.1
WP_011245070.1
WP_060671092.1
ATP39939.1
WP_054612421.1
WP_065294258.1
WP_058299972.1
WP_036126042.1
WP_074094633.1

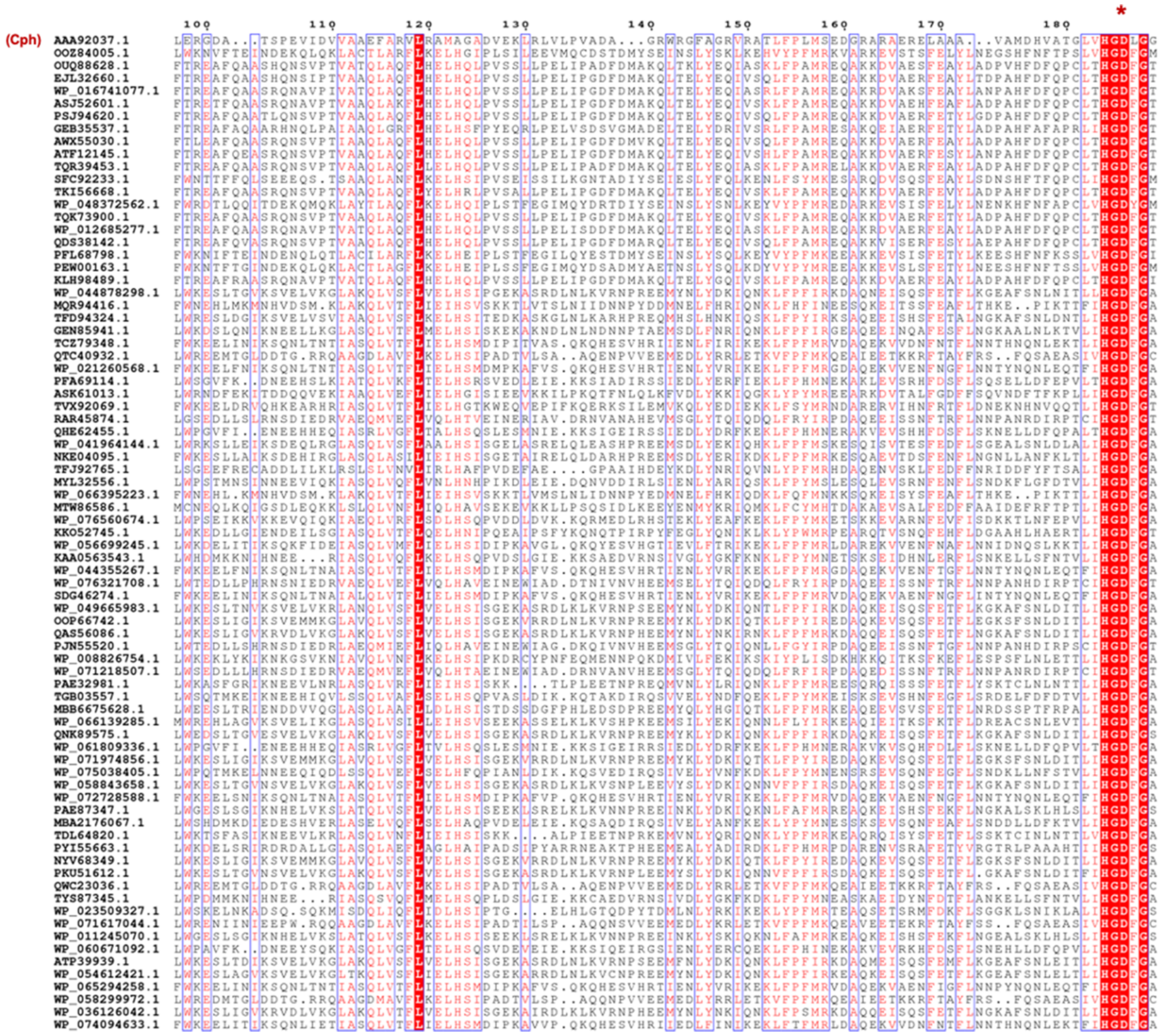

(Cph)
*
100
110
120
130
140
150
160
170
180
AAA92037.1
OOZ84005.1
OUQ88628.1
EJL32660.1
WP_016741077.1
ASJ52601.1
PSJ94620.1
GEB35537.1
AWX55030.1
ATF12145.1
TQR39453.1
SFC92233.1
TKI56668.1
WP_048372562.1
TQK73900.1
WP_012685277.1
QDS38142.1
PFL68798.1
PEW00163.1
KLH98489.1
WP_044878298.1
MQR94416.1
TFD94324.1
GEN85941.1
TCZ79348.1
QTC40932.1
WP_021260568.1
PFA69114.1
ASK61013.1
TVX92069.1
RAR45874.1
QHE62455.1
WP_041964144.1
NKE04095.1
TFJ92765.1
MYL32556.1
WP_066395223.1
MTW86586.1
WP_076560674.1
KKO52745.1
WP_056699245.1
KAA0563543.1
WP_044355267.1
WP_076321708.1
SDG46274.1
WP_049665983.1
OOP66742.1
QAS56086.1
PJN55520.1
WP_008826754.1
WP_071218507.1
PAE32981.1
TGB03557.1
MBB6675628.1
WP_066139285.1
QNK89575.1
WP_061809336.1
WP_071974856.1
WP_075038405.1
WP_058843658.1
WP_072728588.1
PAE87347.1
MBA2176067.1
TDL64820.1
PYI55663.1
NYV68349.1
PKU51612.1
QWC23036.1
TYS87345.1
WP_023509327.1
WP_071617044.1
WP_011245070.1
WP_060671092.1
ATP39939.1
WP_054612421.1
WP_065294258.1
WP_058299972.1
WP_036126042.1
WP_074094633.1

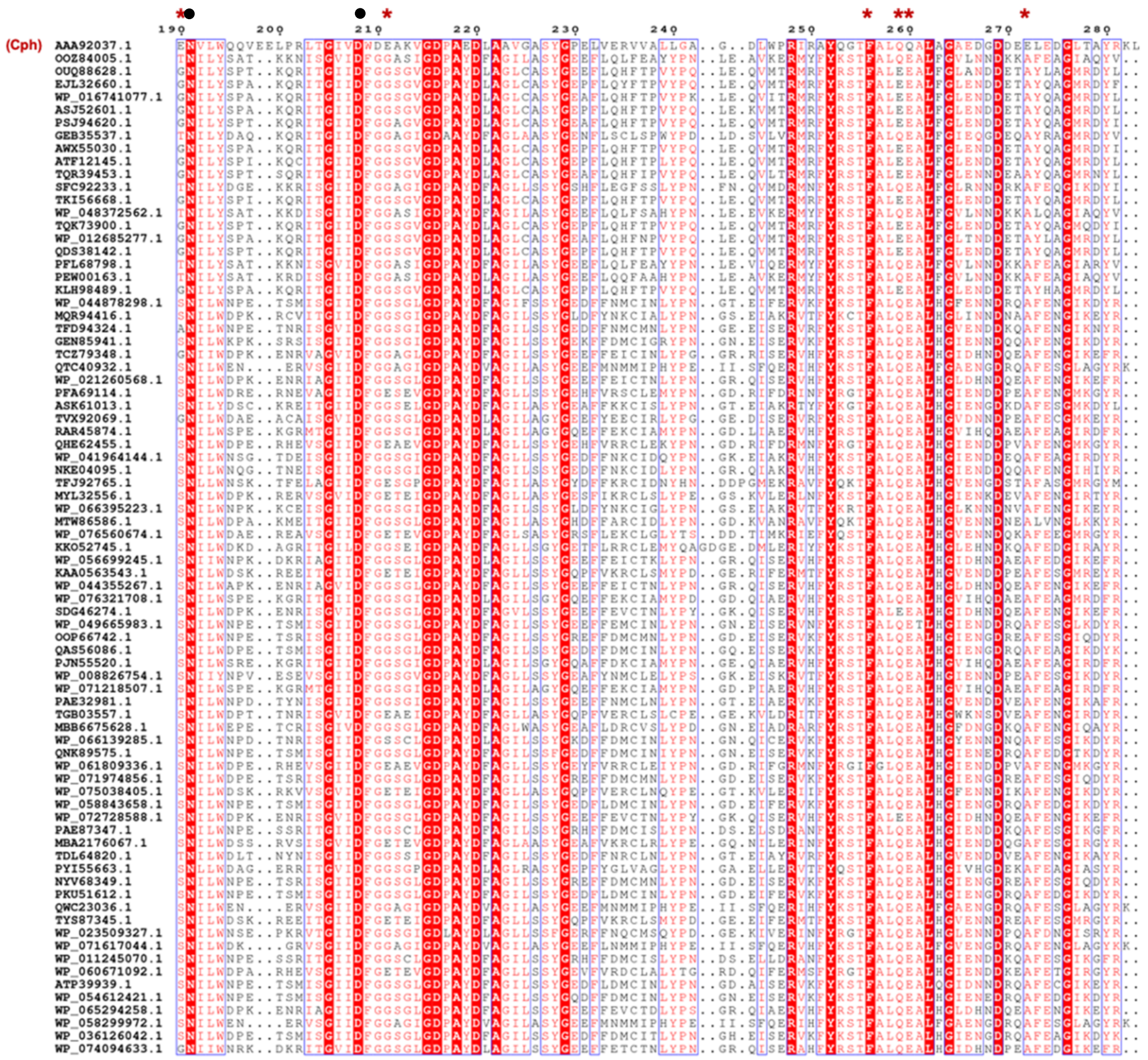

Supplementary Fig. 2 Sequence alignment of Cph with proteins from cluster II. Aligned residues are colored on the bases of the level of conservation (red background shows identity, red character for similarity, and blue frame for similarity across group). The residues involved in CMN binding in Cph are marked with an red asterisk (*) above the sequence alignment. The putative Mg^{2+}-binding residues are marked with a black dot (●) above the sequence alignment.

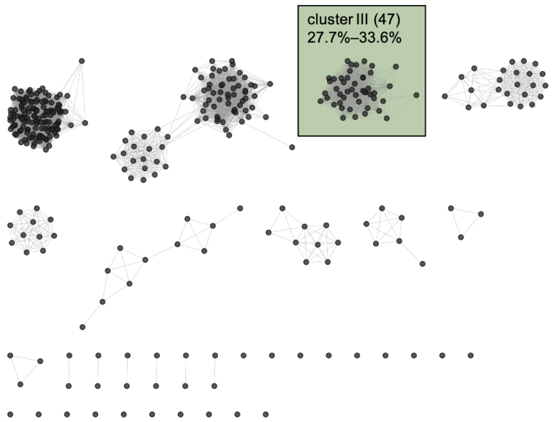
cluster III (47)
27.7%–33.6%

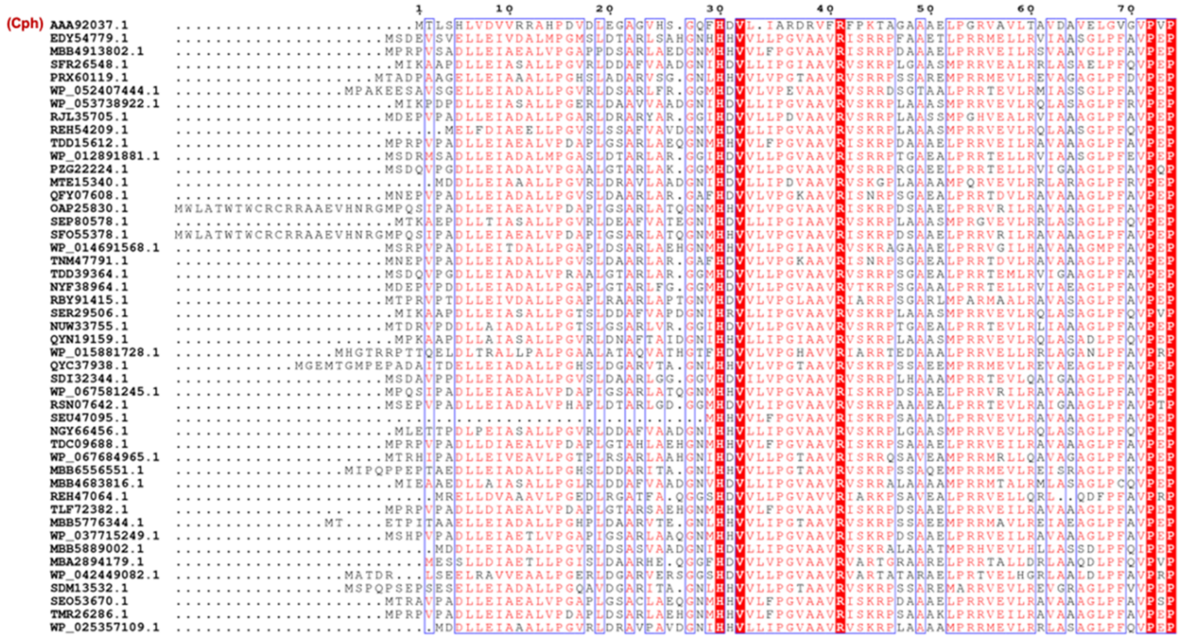
(Cph)

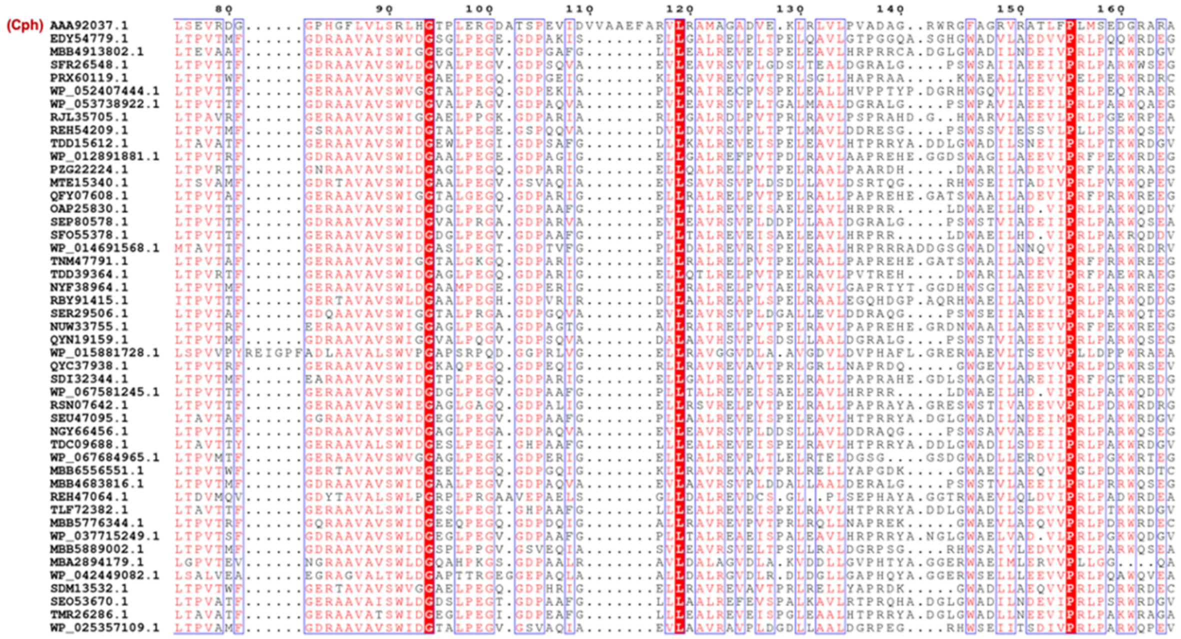
(Cph)

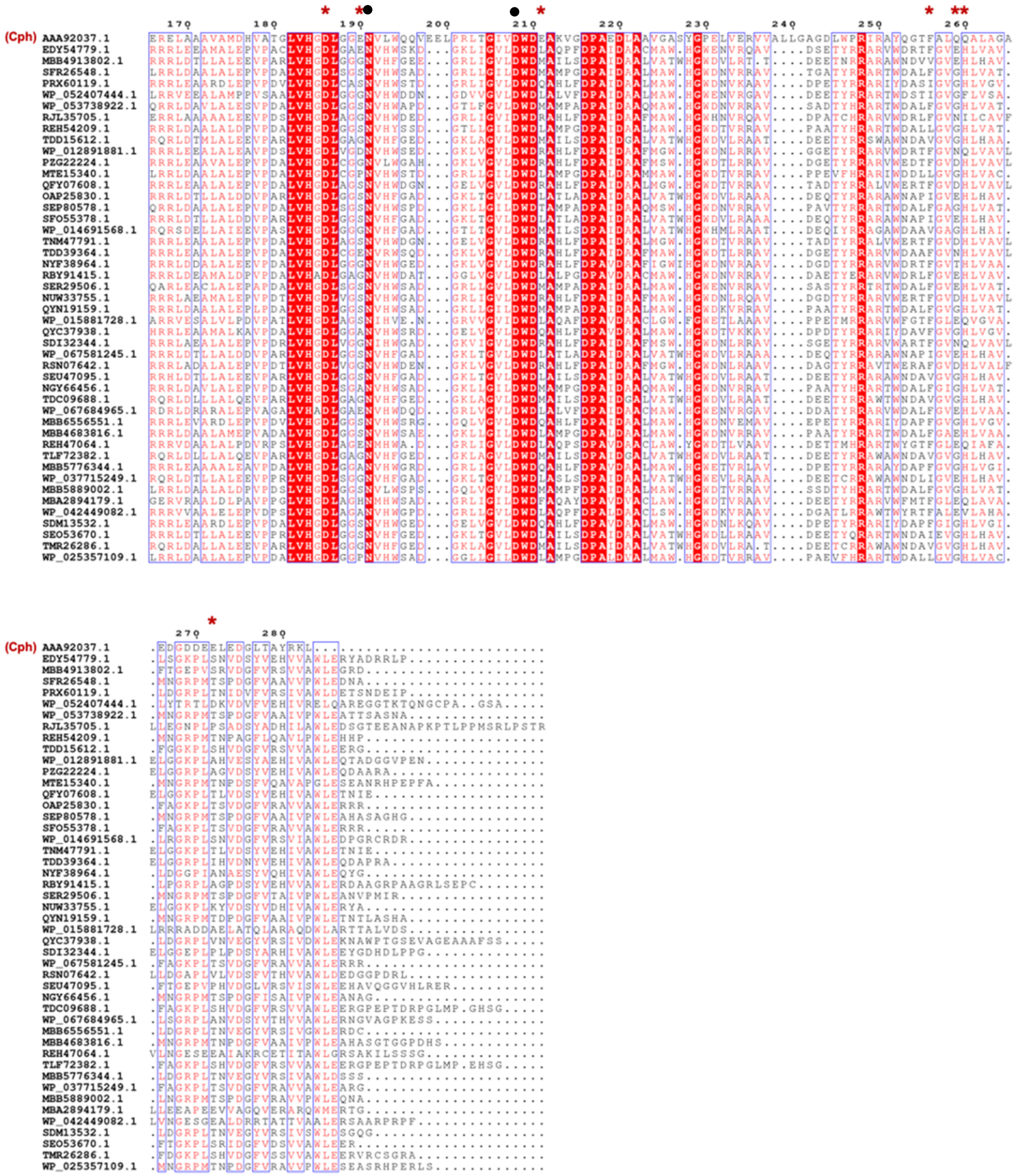

Supplementary Fig. 3 Sequence alignment of Cph with proteins from cluster III. Aligned residues are colored on the bases of the level of conservation (red background shows identity, red character for similarity, and blue frame for similarity across group). The residues involved in CMN binding in Cph are marked with an red asterisk (*) above the sequence alignment. The putative Mg^{2+}-binding residues are marked with a black dot (●) above the sequence alignment.

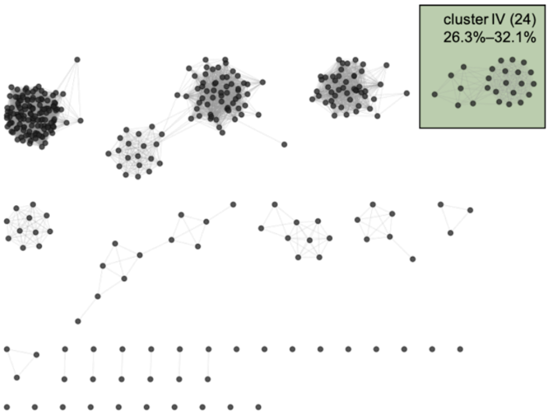
cluster IV (24)
26.3%–32.1%

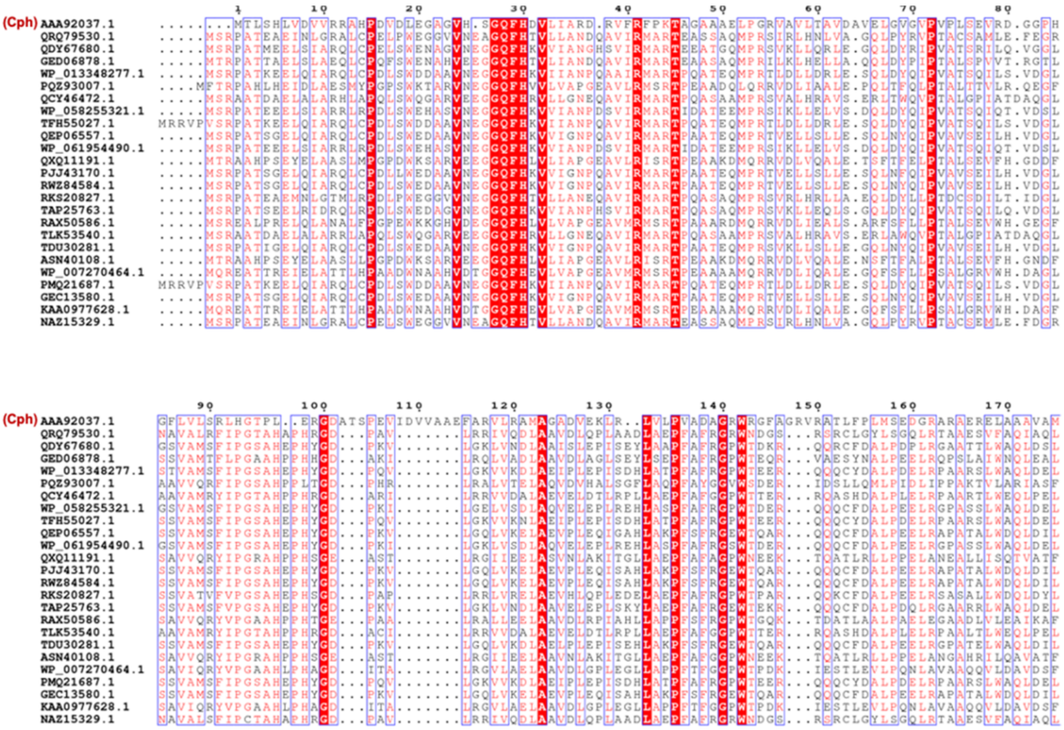
(Cph)
AAA92037.1
QRQ79530.1
QDY67680.1
GED06878.1
WP_013348277.1
PQZ93007.1
QCY46472.1
WP_058255321.1
TFH55027.1
QEP06557.1
WP_061954490.1
QXQ11191.1
PJJ43170.1
RWZ84584.1
RKS20827.1
TAP25763.1
RAX50586.1
TLK53540.1
TDU30281.1
ASN40108.1
WP_007270464.1
PMQ21687.1
GEC13580.1
KAA0977628.1
NAZ15329.1

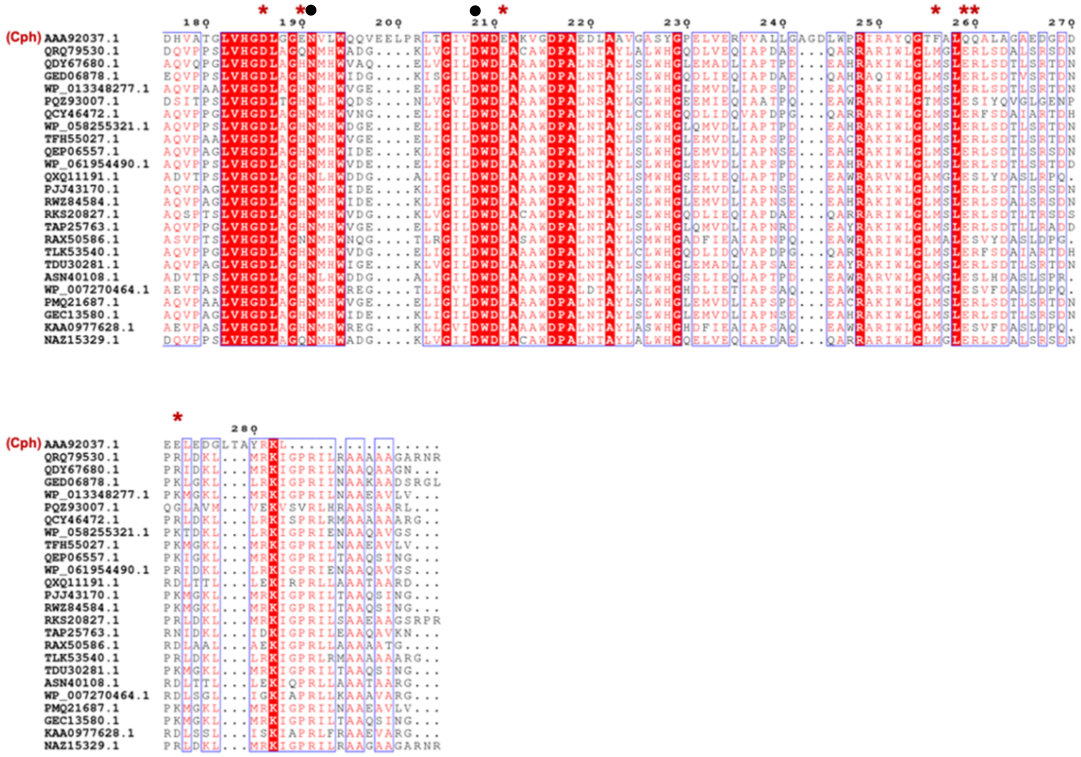

Supplementary Fig. 4 Sequence alignment of Cph with proteins from cluster IV. Aligned residues are colored on the bases of the level of conservation (red background shows identity, red character for similarity, and blue frame for similarity across group). The residues involved in CMN binding in Cph are marked with an red asterisk (*) above the sequence alignment. The putative Mg^{2+}-binding residues are marked with a black dot (●) above the sequence alignment.

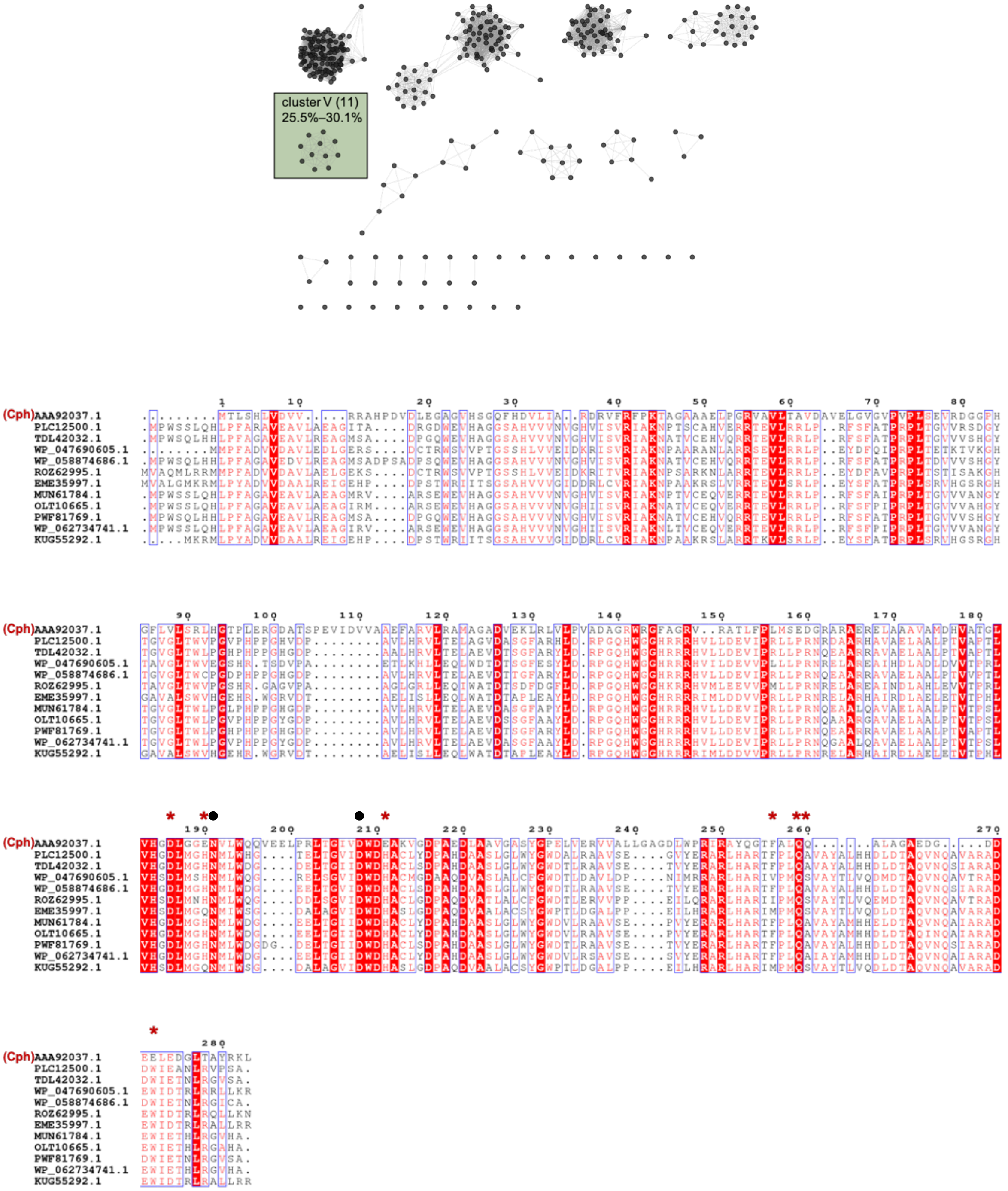

Supplementary Fig. 5 Sequence alignment of Cph with proteins from cluster V. Aligned residues are colored on the bases of the level of conservation (red background shows identity, red character for similarity, and blue frame for similarity across group). The residues involved in CMN binding in Cph are marked with an red asterisk (*) above the sequence alignment. The putative Mg^{2+}-binding residues are marked with a black dot (●) above the sequence alignment.

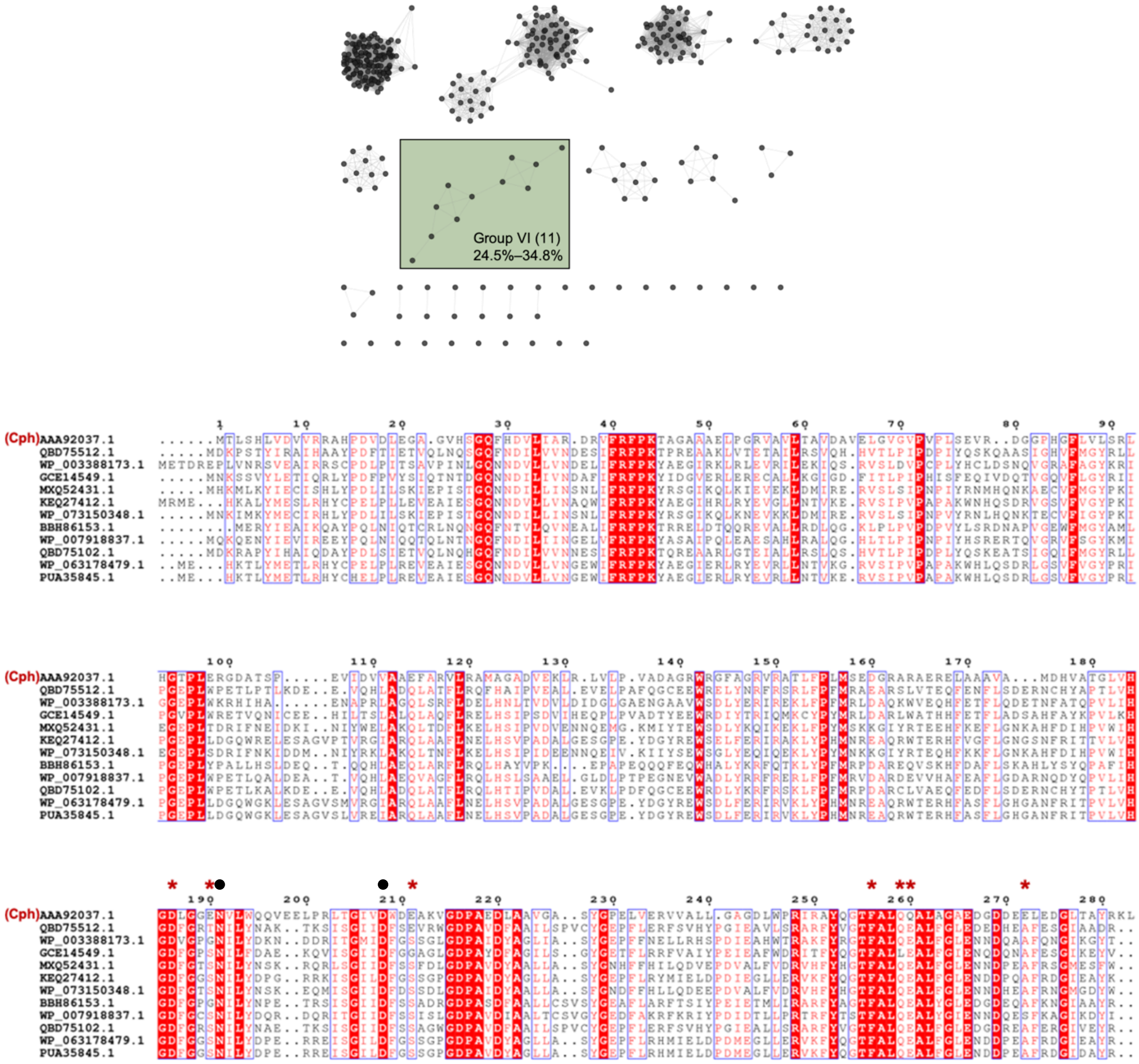

Supplementary Fig. 6 Sequence alignment of Cph with proteins from Group VI. Aligned residues are colored on the bases of the level of conservation (red background shows identity, red character for similarity, and blue frame for similarity across group). The residues involved in CMN binding in Cph are marked with an red asterisk (*) above the sequence alignment. The putative Mg^{2+}-binding residues are marked with a black dot (●) above the sequence alignment.

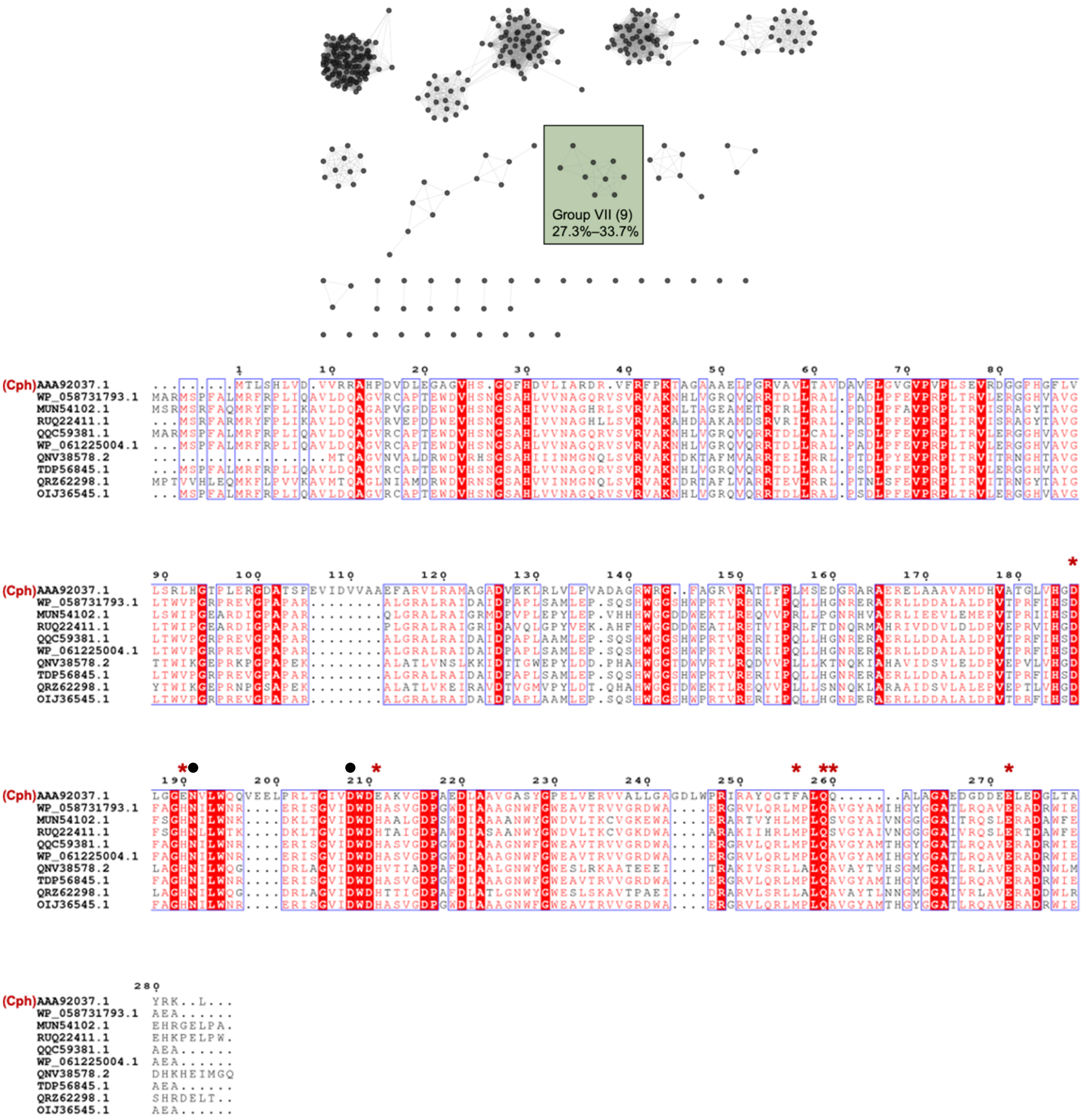

Supplementary Fig. 7 Sequence alignment of Cph with proteins from Group VII. Aligned residues are colored on the bases of the level of conservation (red background shows identity, red character for similarity, and blue frame for similarity across group). The residues involved in CMN binding in Cph are marked with an red asterisk (*) above the sequence alignment. The putative Mg^{2+}-binding residues are marked with a black dot (●) above the sequence alignment.

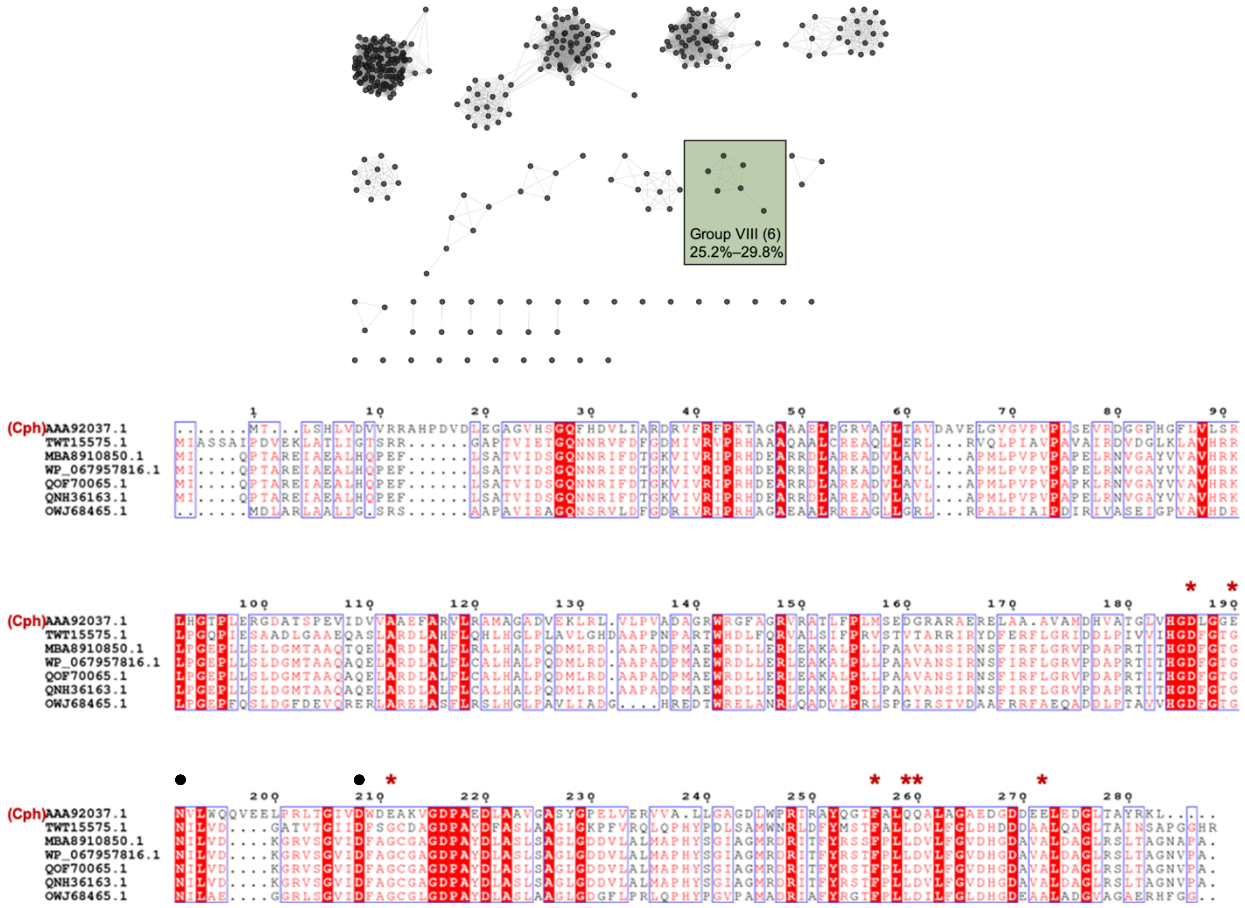

Supplementary Fig. 8 Sequence alignment of Cph with proteins from Group VIII. Aligned residues are colored on the bases of the level of conservation (red background shows identity, red character for similarity, and blue frame for similarity across group). The residues involved in CMN binding in Cph are marked with an red asterisk (*) above the sequence alignment. The putative Mg^{2+}-binding residues are marked with a black dot (●) above the sequence alignment.

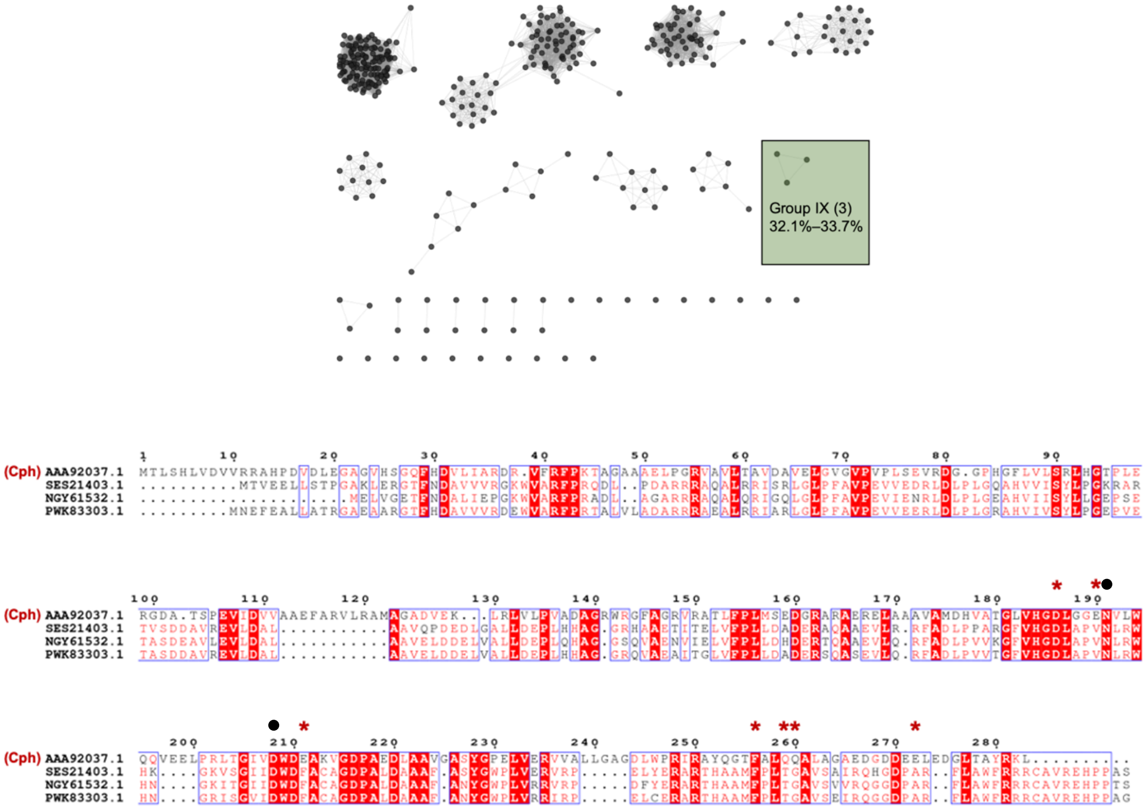

Supplementary Fig. 9 Sequence alignment of Cph with proteins from Group IX. Aligned residues are colored on the bases of the level of conservation (red background shows identity, red character for similarity, and blue frame for similarity across group). The residues involved in CMN binding in Cph are marked with an red asterisk (*) above the sequence alignment. The putative Mg^{2+}-binding residues are marked with a black dot (●) above the sequence alignment.

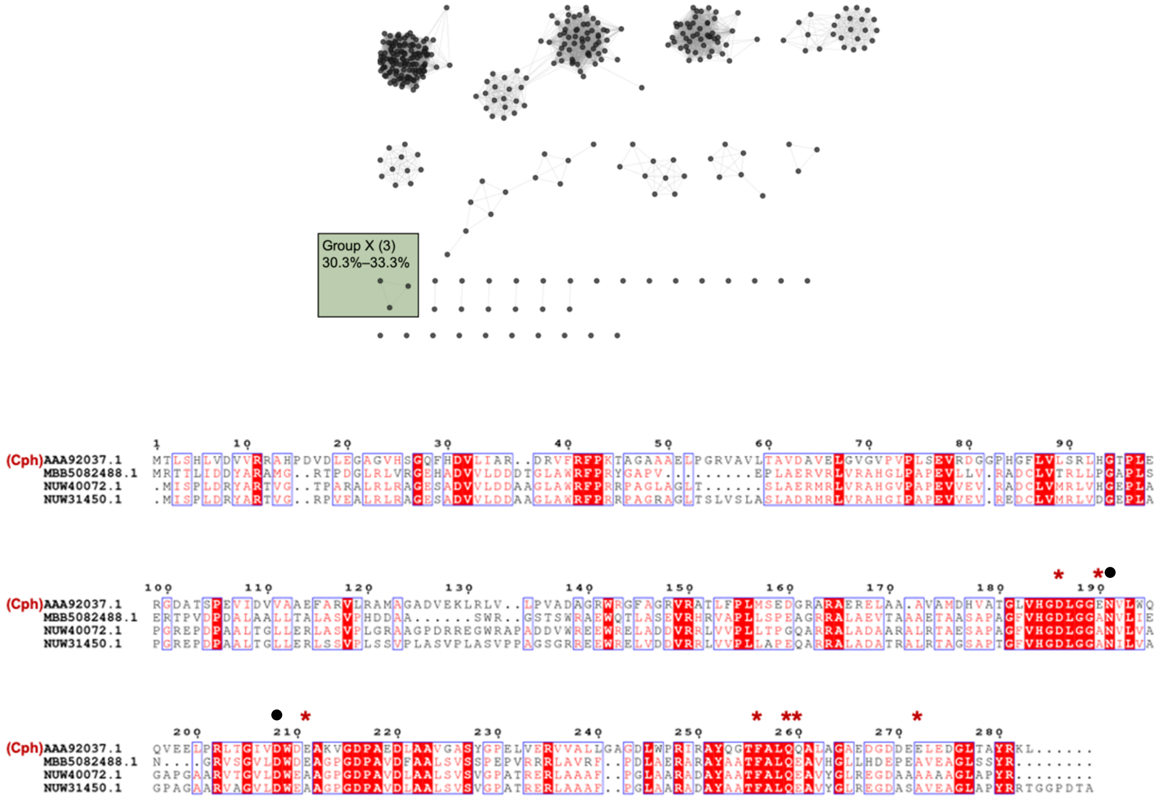

Supplementary Fig. 10 Sequence alignment of Cph with proteins from Group X. Aligned residues are colored on the bases of the level of conservation (red background shows identity, red character for similarity, and blue frame for similarity across group). The residues involved in CMN binding in Cph are marked with an red asterisk (*) above the sequence alignment. The putative Mg^{2+}-binding residues are marked with a black dot (●) above the sequence alignment.

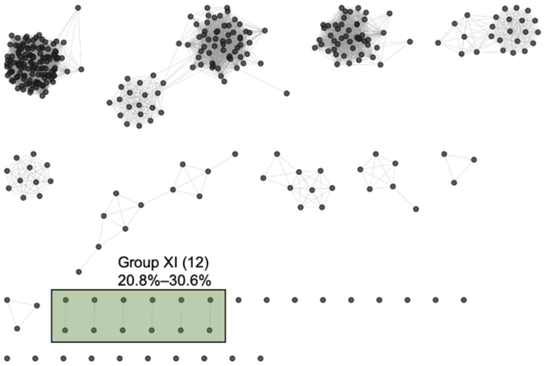

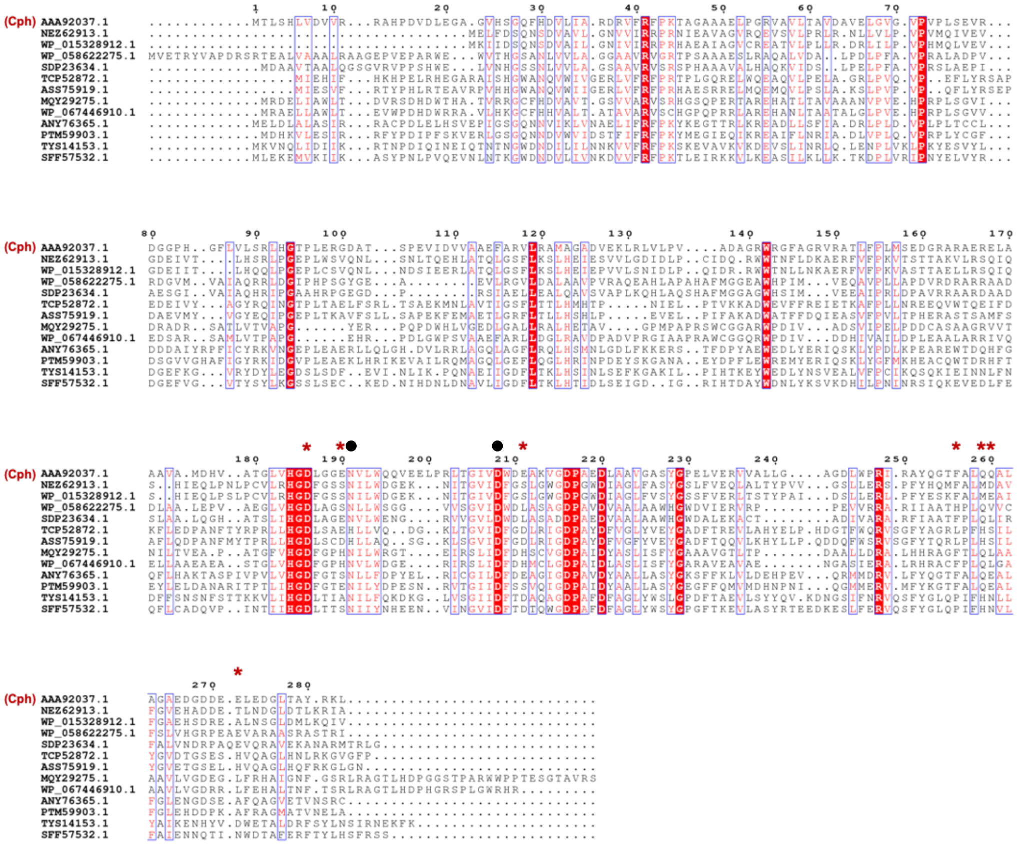

Supplementary Fig. 11 Sequence alignment of Cph with proteins from Group XI. Aligned residues are colored on the bases of the level of conservation (red background shows identity, red character for similarity, and blue frame for similarity across group). The residues involved in CMN binding in Cph are marked with an red asterisk (*) above the sequence alignment. The putative Mg^{2+}-binding residues are marked with a black dot (●) above the sequence alignment.

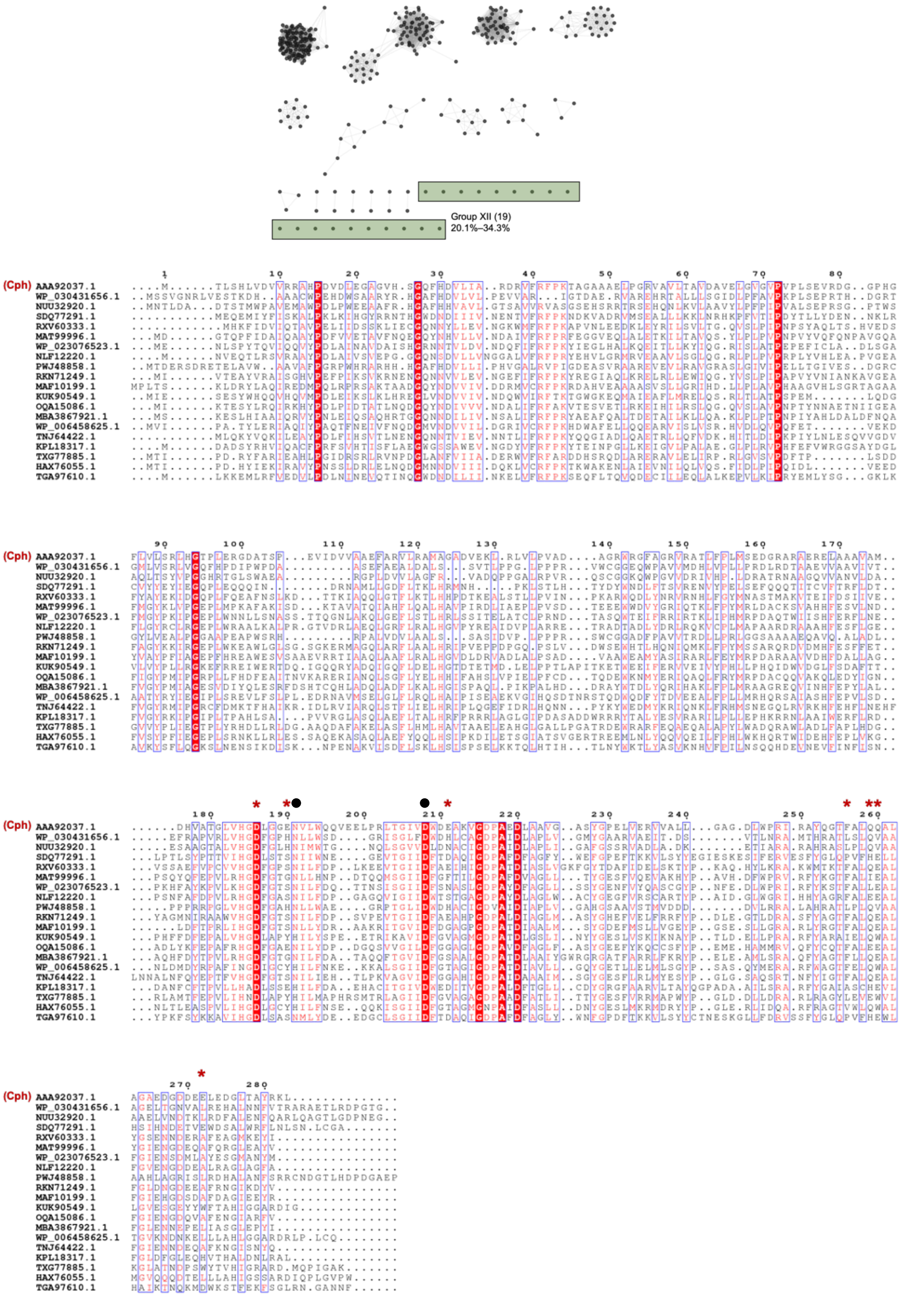

Supplementary Fig. 12 Sequence alignment of Cph with proteins from Group XII. Aligned residues are colored on the bases of the level of conservation (red background shows identity, red character for similarity, and blue frame for similarity across group). The residues involved in CMN binding in Cph are marked with an red asterisk (*) above the sequence alignment. The putative Mg^{2+}-binding residues are marked with a black dot (●) above the sequence alignment.

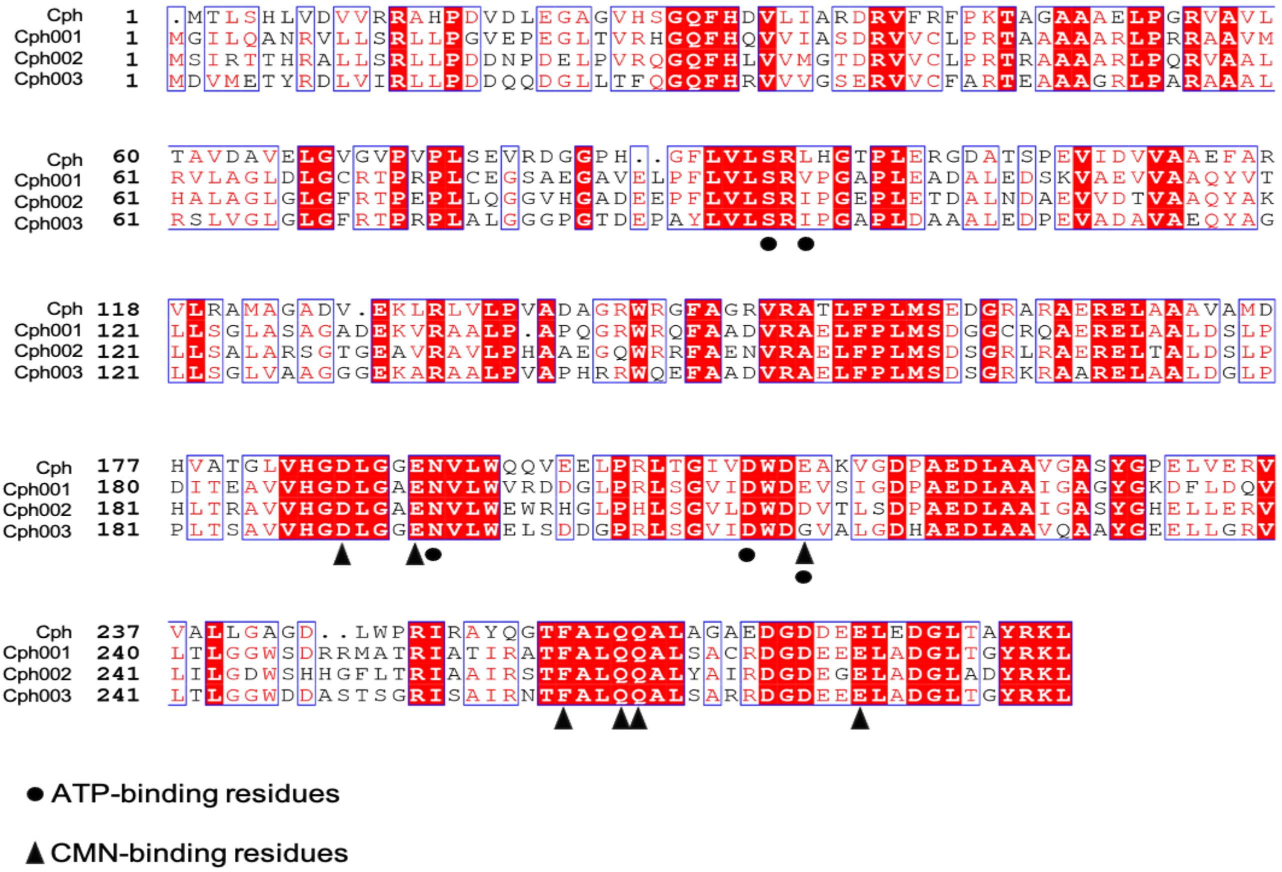

Supplementary Fig. 13 Sequence alignment of Cph with its homologues.
Aligned residues are colored on the bases of the level of conservation (red background shows identity, red character for similarity, and blue frame for similarity across group). The residues involved in ATP and CMN binding are labeled under the sequence alignment by ● and ▲, respectively.

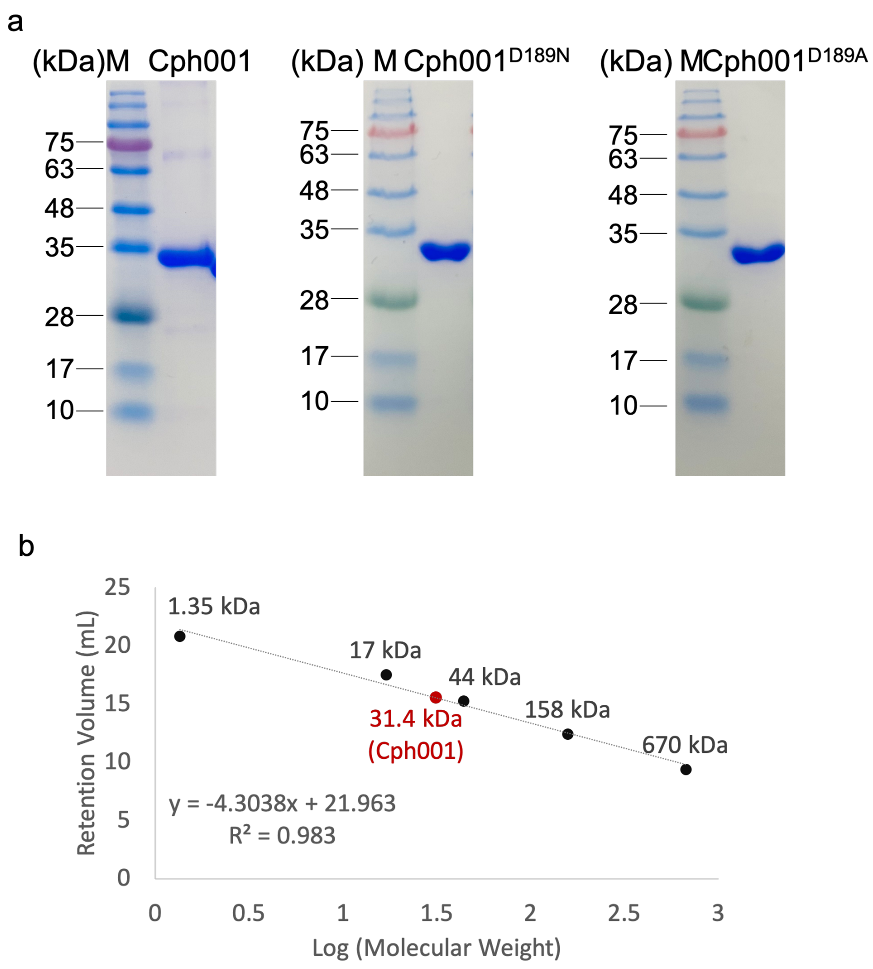

Supplementary Fig. 14 SDS-PAGE and molecular weight estimation of Cph001.
a. SDS-PAGE of Cph001 and the mutant Cph001^{D189N} and Cph001^{D189A}. The calculated molecular weight of Cph001 is ~32.5 kDa. **b.** Molecular weight estimation of Cph001 by size exclusion chromatography. The size exclusion chromatography was carried out on a Superdex 200 10/300 GL column (Cytiva). The apparent molecule weight of Cph001 is estimated to be 31.4 kDa, suggesting that Cph001 is monomer in solution.

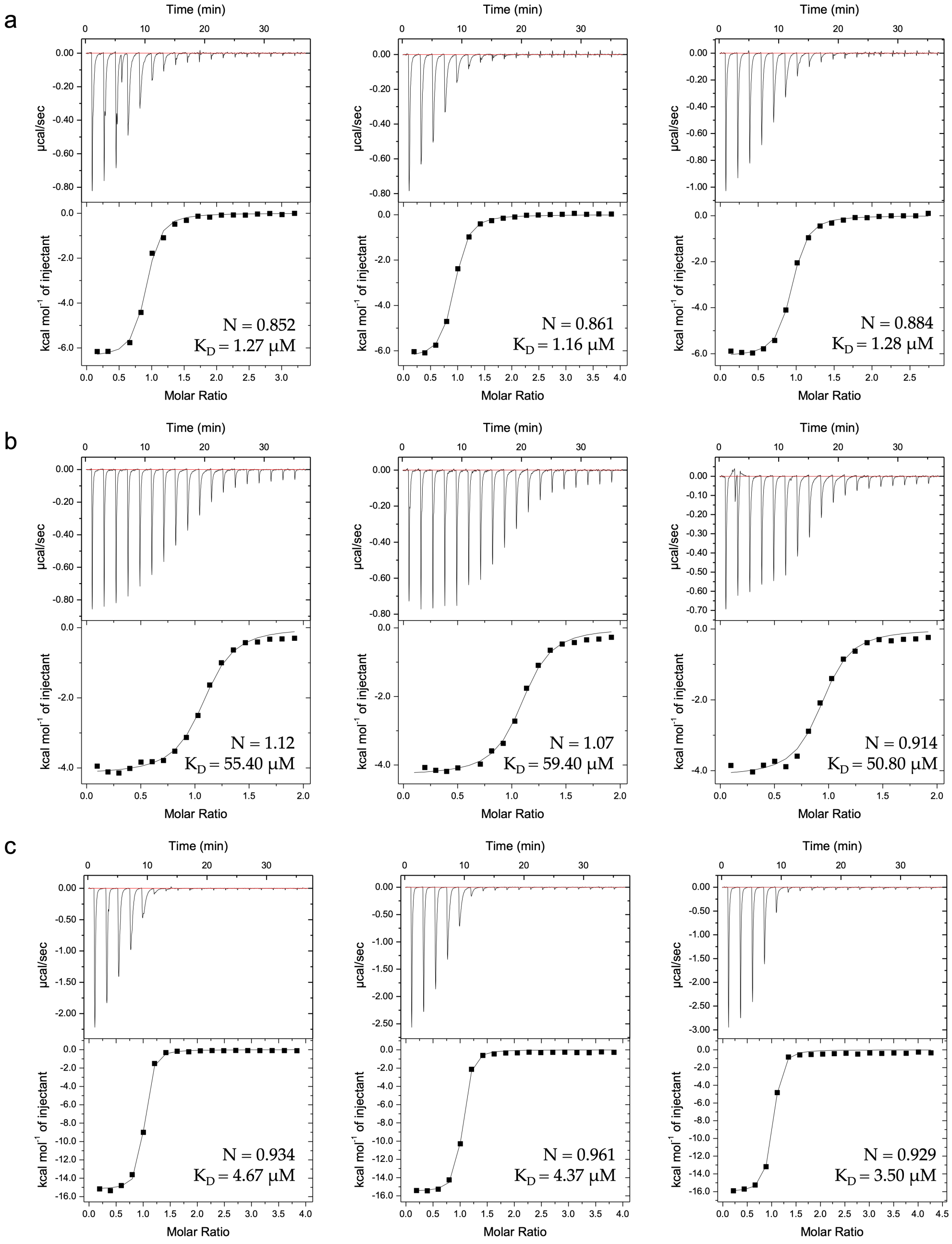

Supplementary Fig. 15 ITC analysis of the protein-ligand binding interaction.

Data are shown for the binding of **a.** CMN IIA, **b.** CMN IIB, and **c.** VIO against Cph001. Lower panel of each figure shows the fit to the binding curve with resulting dissociation constants (K_D) and stoichiometries (N). All reactions were performed in triplicate.

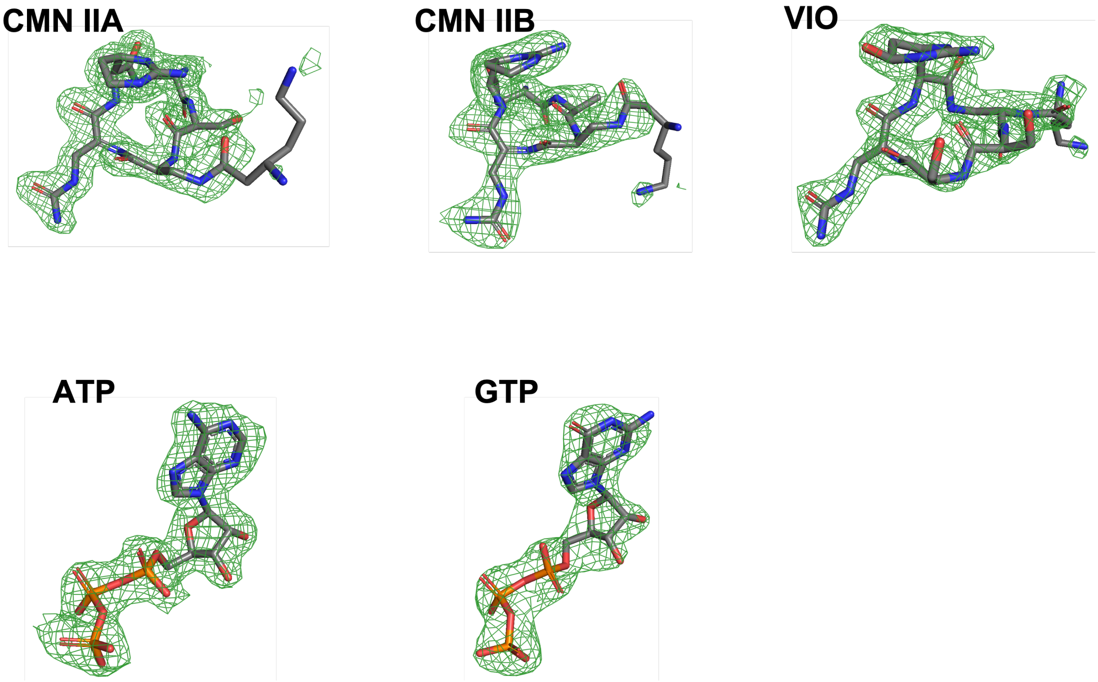

Supplementary Fig. 16 The electron density maps of CMN IIA, CMN IIB, VIO, ATP, and GTP.

The composite ($2mF_o - DF_c$) omit maps of CMN IIA, CMN IIB, VIO, ATP, and GTP in the complex structures Cph001^{D189N}-CMN IIA, Cph001^{D189N}-CMN IIB, Cph001^{D189N}-VIO, Cph001^{D189N}-ATP, and Cph001^{D189N}-GTP, respectively, colored green were contoured at 1.0 σ.

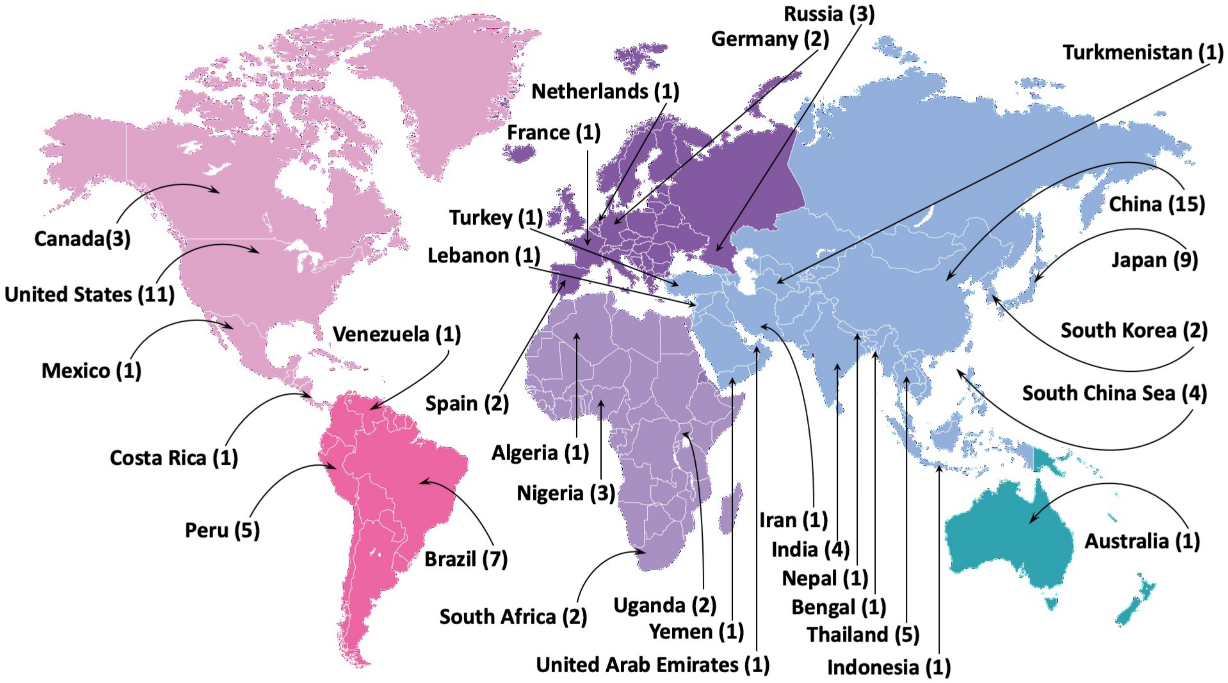

Supplementary Fig. 17 Geographic distribution of the proteins in cluster I. (Supplementary Table 1 for details).

Supplementary Table 1 The selected homologues in the cluster 1

Strain	Location	Protein ID	Amino Acid Sequence Identity (%)
Saccharothrix mutabilis subsp. *Capreolus* (Cph)	South China Sea	AAA92037	100
Streptosporangium roseum (Cph001)	United States	WP_012891209	49.02
Streptomyces sp. SID8364 (Cph002)	-	MYT78782	48.16
Streptomyces filamentosus NRRL 11379 (Cph003)	-	WP_006126679	45.17
Actinomadura bangladeshensis	Bengal	NEA24112	45.68
Actinomadura chibensis	Japan	WP_067895405	45.35
Actinomadura decatromicini	Thailand	TYK51016	47.04
Actinomadura geliboluensis	Turkey	TMR41404	51.25
Actinomadura litoris	China	MUN39125	44.32
Actinomadura luteofluorescens	Japan	NYD47829	44.32
Actinomadura montaniterrae	Thailand	KAB2390512	45.19
Actinomadura pelletieri DSM 43383	Nigeria	RKS79035	41.35
Actinomadura sp. LHW52907	South China Sea	RFS87215	40.69
Actinomadura sp. NAK00032	China	QKW35503	50.58
Actinomadura sp. WAC 06369	Nigeria	RSN70164	40.98
Actinomadura syzygii	Thailand	TYC15423	44.14
Allostreptomyces psammosilenae	China	NYI03613	46.54
Marinactinospora thermotolerans DSM 45154	South China Sea	SJZ91120	46.38
Mycobacterium porcinum	-	WP_036447945	42.58
Mycobacterium sp. 455mf	-	SFF63122	42.09
Mycobacterium vulneris	Netherlands	WP_065457399	42.13
Mycolicibacterium boenickei	United States	QRY45733	42.6
Mycolicibacterium fortuitum	Brazil	ALI24257	31.49
Mycolicibacterium fortuitum	Brazil	NOP96578	41.76
Mycolicibacterium fortuitum	Brazil	NOQ60147	41.76
Mycolicibacterium fortuitum	Brazil	OBK11373	42.23
Mycolicibacterium fortuitum	Brazil	EJZ15587	44.64
Mycolicibacterium peregrinum	Uganda	TGB36013	42.26
Mycolicibacterium peregrinum	Uganda	WP_064879567	43.61
Mycolicibacterium septicum	-	QRY51388	41.9
Mycolicibacterium sp. CBMA 247	Brazil	MUM38776	43.62

Mycolicibacterium wolinskyi	United States	ORX09226	43.99
Nocardia arthritidis	Australia	QIS08454	39.73
Nocardia iowensis	United States	QXN95299	47.24
Nocardia ninae NBRC 108245	France	GEM43105	39.58
Nocardia panacis	China	RJO73356	45.47
Nocardia puris		WP_067501823	41.72
Nocardia sp. CS682	Republic of Korea	QBS46129	36.90
Nocardia speluncae	Republic of Korea	WP_068036065	49.37
Nocardia tenerifensis	Spain	PXX67008	40.18
Nocardia vulneris	United States	KIA61878	39.31
Nocardiopsis alba	United States	MYR35384	43.03
Nonomuraea jabiensis	Nigeria	MBB5782999	50.41
Nonomuraea rubra		MBB6550120	42.83
Nonomuraea solani	China	SEG44111	45.26
Nonomuraea soli	China	MBA2897536	43.01
Nonomuraea sp. ATCC 55076	India	SPL99404	46.52
Nonomuraea sp. C10	Nepal	TXK42967	42.58
Nonomuraea sp. WAC 01424	Canada	RSN09352	45.12
Pseudonocardia sp. HH130630-07	Costa Rica	WP_068800897	42.68
Saccharothrix australiensis	Australia	RKT55734	57.31
Saccharothrix espanaensis	Spain	CCH33049	61.92
Saccharothrix tamanrassetensis	Algeria	MBB5957136	68.9
Saccharothrix variisporea	India	RKT74338	83.99
Sphaerisporangium krabiense	Thailand	MBB5630345	45.93
Spongiactinospora gelatinilytica	Turkmenistan	PZG51559	45.7
Spongiactinospora rosea	South China Sea	RBQ20497	44.15
Streptoalloteichus hindustanus	India	SHF88622	45.64
Streptomyces acidiscabies	United States	WP_040839000	44.65
Streptomyces alfalfae	China	QQC94128	45.85
Streptomyces anulatus	Finland	QYA92321	42.28
Streptomyces anulatus	Finland	NEC00960	42.67
Streptomyces anulatus	Finland	NEB82822	43.81
Streptomyces atratus	Japan	WP_072486304	45.85
Streptomyces badius	Russia	WP_030815486	46.38
Streptomyces buecherae	United States	QKW52403	44.34
Streptomyces buecherae	United States	QNJ40085	45.22
Streptomyces globisporus C-1027	China	WP_010064695	45

Streptomyces griseostramineus	Russia	MBB4902825	46.03
Streptomyces griseus subsp. *Griseus*	Japan	WP_012377782	47.29
Streptomyces libani subsp. *Libani*	Lebanon	GFE27010	43.46
Streptomyces luteoverticillatus	China	AZQ75066	45.63
Streptomyces lydicus	United States	AZS69952	43.46
Streptomyces nanshensis	South China Sea	WP_070199995	47.83
Streptomyces niveus NCIMB 11891	United States	WP_023540635	44.15
Streptomyces palmae	Thailand	TGB02546	45.86
Streptomyces parvus		TYR51927	44.83
Streptomyces parvus		KAA6198520	44.83
Streptomyces rhizosphaericus	Indonesia	NEW75133	45.34
Streptomyces rubrolavendulae		WP_069980007	47.22
Streptomyces sp. 11-1-2	Canada	ASQ93368	45
Streptomyces sp. AgN23		QTI90402	45.69
Streptomyces sp. CAI 127		NUW04279	45.17
Streptomyces sp. CB00072		WP_073869800	44.51
Streptomyces sp. CB00271	China	WP_073803518	44.85
Streptomyces sp. CB01580	China	OKJ28143	44.84
Streptomyces sp. CB02115		WP_073743786	44.33
Streptomyces sp. CB02366	United Arab Emirates	WP_073766824	45.68
Streptomyces sp. CB02613		PJN34974	44.49
Streptomyces sp. CB04723	China	QLG30641	48.59
Streptomyces sp. CRXT-G-22	China	QNP75422	44.53
Streptomyces sp. CS014	United States	PVC99054	44.83
Streptomyces sp. CS057	Peru	OWA15020	43.71
Streptomyces sp. CS090A		PVC80687	45
Streptomyces sp. CS131		PVC82821	45.17
Streptomyces sp. CS147		PVD00169	45.17
Streptomyces sp. HCCB10043		ESU47527	47.47
Streptomyces sp. MMG1522	United States	WP_030340787	48.25
Streptomyces sp. MNU77	India	WP_047175012	44.05
Streptomyces sp. NBS 14/10	Brazil	OXL33712	50.58
Streptomyces sp. NRRL S-1521		WP_062780735	46.38
Streptomyces sp. PBH53	Canada	AKN74990	49.2
Streptomyces sp. Rer75	China	QLH20617	44.66
Streptomyces sp. Root63	Germany	WP_056708000	44

Streptomyces sp. SID3343		MYV98955	42.33
Streptomyces sp. SID4951		MYT18546	45.17
Streptomyces sp. SID6648		NED02447	45.17
Streptomyces sp. SID8352		MYU21225	45.52
Streptomyces sp. SID8369	South Africa	MYW78241	47.91
Streptomyces sp. SID8378		MYX04840	45.17
Streptomyces sp. sk2.1		TXS71028	45.34
Streptomyces sp. *Termitarium*-T10T-6		SCD90189	45.17
Streptomyces sp. TSRI0281		WP_073719976	45.68
Streptomyces sp. TSRI0395		WP_073960519	44
Streptomyces sp. TYQ1024		MBC2876542	44.49
Streptomyces sp. V2	Mexico	WP_055724205	45.13
Streptomyces sp. W007	China	WP_007458495	43.66
Streptomyces sp. WAC05858		RSS42509	45.68
Streptomyces sp. ZL-24		POG47903	47.49
Streptomyces tubercidicus	Japan	GFE39783	45.51
Streptomyces venezuelae	Venezuela	QES47477	47.87
Streptomyces vinaceus		AAP92507	47.18
Streptomyces vinaceus		WP_030275963	48.25
Streptomyces violaceusniger	Yemen	WP_059147333	45.68
Streptomyces vitaminophilus	Japan	WP_018381717	43.81
Streptomyces zagrosensis	Iran	MBB5935728	46.04
Streptosporangium album	Japan	MBB4943325	46.03
Streptosporangium minutum	South Africa	OUC86915	47.32
Streptosporangium sandarakinum	Germany	NYF44603	45.68
Thermomonospora echinospora	Japan	SEG81901	47.37
