# Supplementary material for: Discovery and characterization of genes conferring natural resistance to the antituberculosis antibiotic capreomycin
Source: Commun Biol. 2023 Dec 19;6:1282. doi: 10.1038/s42003-023-05681-6 (PMC10730852; doi:10.1038/s42003-023-05681-6)
Supplement: Supplementary file 3 — Description of Additional Supplementary Files [file 42003_2023_5681_MOESM3_ESM.docx]

Description of Additional Supplementary Files

**File name:** Supplementary Data 1

**Description:** The source data of Figure 3b, Figure 3c, and Supplementary Figure 14b
