# Supplementary material for: Discovery and characterization of genes conferring natural resistance to the antituberculosis antibiotic capreomycin
Source: Commun Biol. 2023 Dec 19;6:1282. doi: 10.1038/s42003-023-05681-6 (PMC10730852; doi:10.1038/s42003-023-05681-6)
Supplement: Supplementary file 5 — Reporting Summary [file 42003_2023_5681_MOESM5_ESM.pdf]

nature portfolio

Corresponding author(s): Chin-Yuan Chang

Last updated by author(s): Nov 29, 2023

Reporting Summary

Nature Portfolio wishes to improve the reproducibility of the work that we publish. This form provides structure for consistency and transparency in reporting. For further information on Nature Portfolio policies, see our Editorial Policies and the Editorial Policy Checklist.

Statistics

For all statistical analyses, confirm that the following items are present in the figure legend, table legend, main text, or Methods section.

n/a	Confirmed	
☐	☒	The exact sample size (*n*) for each experimental group/condition, given as a discrete number and unit of measurement
☒	☐	A statement on whether measurements were taken from distinct samples or whether the same sample was measured repeatedly
☒	☐	The statistical test(s) used AND whether they are one- or two-sided<br>*Only common tests should be described solely by name; describe more complex techniques in the Methods section.*
☒	☐	A description of all covariates tested
☒	☐	A description of any assumptions or corrections, such as tests of normality and adjustment for multiple comparisons
☐	☒	A full description of the statistical parameters including central tendency (e.g. means) or other basic estimates (e.g. regression coefficient) AND variation (e.g. standard deviation) or associated estimates of uncertainty (e.g. confidence intervals)
☒	☐	For null hypothesis testing, the test statistic (e.g. *F*, *t*, *r*) with confidence intervals, effect sizes, degrees of freedom and *P* value noted<br>*Give P values as exact values whenever suitable.*
☒	☐	For Bayesian analysis, information on the choice of priors and Markov chain Monte Carlo settings
☒	☐	For hierarchical and complex designs, identification of the appropriate level for tests and full reporting of outcomes
☒	☐	Estimates of effect sizes (e.g. Cohen's *d*, Pearson's *r*), indicating how they were calculated

Our web collection on statistics for biologists contains articles on many of the points above.

Software and code

Policy information about availability of computer code

Data collection	HKL2000
Data analysis	COOT, REFMAC5, GraphPad Prism 5, Cytoscape 3.9.1, and WebLogo 3.

For manuscripts utilizing custom algorithms or software that are central to the research but not yet described in published literature, software must be made available to editors and reviewers. We strongly encourage code deposition in a community repository (e.g. GitHub). See the Nature Portfolio guidelines for submitting code & software for further information.

Data

Policy information about availability of data

All manuscripts must include a data availability statement. This statement should provide the following information, where applicable:

- Accession codes, unique identifiers, or web links for publicly available datasets
- A description of any restrictions on data availability
- For clinical datasets or third party data, please ensure that the statement adheres to our policy

Protein Data Bank with accession codes 8I80[http://doi.org/10.2210/pdb8I80/pdb], 8I82[http://doi.org/10.2210/pdb8I82/pdb], 8I84[http://doi.org/10.2210/pdb8I84/pdb], 8I85[http://doi.org/10.2210/pdb8I85/pdb], 8I86[http://doi.org/10.2210/pdb8I86/pdb], 8I89[http://doi.org/10.2210/pdb8I89/pdb], 8I8G[http://doi.org/10.2210/pdb8I8G/pdb], and 8I8H[http://doi.org/10.2210/pdb8I8H/pdb].

Research involving human participants, their data, or biological material

Policy information about studies with human participants or human data. See also policy information about sex, gender (identity/presentation), and sexual orientation and race, ethnicity and racism.

Reporting on sex and gender	not applicable
Reporting on race, ethnicity, or other socially relevant groupings	not applicable
Population characteristics	not applicable
Recruitment	not applicable
Ethics oversight	not applicable

Note that full information on the approval of the study protocol must also be provided in the manuscript.

Field-specific reporting

Please select the one below that is the best fit for your research. If you are not sure, read the appropriate sections before making your selection.

☒ Life sciences ☐ Behavioural & social sciences ☐ Ecological, evolutionary & environmental sciences

For a reference copy of the document with all sections, see nature.com/documents/nr-reporting-summary-flat.pdf

Life sciences study design

All studies must disclose on these points even when the disclosure is negative.

Sample size	All the protein gel filtration experiments have been replicated 3 times and the elution time of proteins are the same. The enzymatic kinetic experiment has three times independent repeats.
Data exclusions	no data exclusions
Replication	We have confirmed the assays can be independently repeated.
Randomization	This is no direct relevance to the study.
Blinding	This is no direct relevance to the study.

Behavioural & social sciences study design

All studies must disclose on these points even when the disclosure is negative.

Study description	This is no direct relevance to the study.
Research sample	This is no direct relevance to the study.
Sampling strategy	This is no direct relevance to the study.
Data collection	This is no direct relevance to the study.
Timing	This is no direct relevance to the study.
Data exclusions	This is no direct relevance to the study.
Non-participation	This is no direct relevance to the study.
Randomization	This is no direct relevance to the study.

Ecological, evolutionary & environmental sciences study design

All studies must disclose on these points even when the disclosure is negative.

Study description	This is no direct relevance to the study.
Research sample	This is no direct relevance to the study.
Sampling strategy	This is no direct relevance to the study.
Data collection	This is no direct relevance to the study.
Timing and spatial scale	This is no direct relevance to the study.
Data exclusions	This is no direct relevance to the study.
Reproducibility	This is no direct relevance to the study.
Randomization	This is no direct relevance to the study.
Blinding	This is no direct relevance to the study.

Did the study involve field work? ☐ Yes ☒ No

Field work, collection and transport

Field conditions	This is no direct relevance to the study.
Location	This is no direct relevance to the study.
Access & import/export	This is no direct relevance to the study.
Disturbance	This is no direct relevance to the study.

Reporting for specific materials, systems and methods

We require information from authors about some types of materials, experimental systems and methods used in many studies. Here, indicate whether each material, system or method listed is relevant to your study. If you are not sure if a list item applies to your research, read the appropriate section before selecting a response.

Materials & experimental systems

n/a	Involved in the study	
☒	☐	Antibodies
☒	☐	Eukaryotic cell lines
☒	☐	Palaeontology and archaeology
☒	☐	Animals and other organisms
☒	☐	Clinical data
☒	☐	Dual use research of concern
☒	☐	Plants

Methods

n/a	Involved in the study	
☒	☐	ChIP-seq
☒	☐	Flow cytometry
☒	☐	MRI-based neuroimaging

Antibodies

Antibodies used	This is no direct relevance to the study.
Validation	This is no direct relevance to the study.

Eukaryotic cell lines

Policy information about cell lines and Sex and Gender in Research

Cell line source(s)	This is no direct relevance to the study.
Authentication	This is no direct relevance to the study.
Mycoplasma contamination	This is no direct relevance to the study.
Commonly misidentified lines (See ICLAC register)	This is no direct relevance to the study.

Palaeontology and Archaeology

Specimen provenance	This is no direct relevance to the study.
Specimen deposition	This is no direct relevance to the study.
Dating methods	This is no direct relevance to the study.

☐ Tick this box to confirm that the raw and calibrated dates are available in the paper or in Supplementary Information.

Ethics oversight	This is no direct relevance to the study.

Note that full information on the approval of the study protocol must also be provided in the manuscript.

Animals and other research organisms

Policy information about studies involving animals; ARRIVE guidelines recommended for reporting animal research, and Sex and Gender in Research

Laboratory animals	This is no direct relevance to the study.
Wild animals	This is no direct relevance to the study.
Reporting on sex	This is no direct relevance to the study.
Field-collected samples	This is no direct relevance to the study.
Ethics oversight	This is no direct relevance to the study.

Note that full information on the approval of the study protocol must also be provided in the manuscript.

Clinical data

Policy information about clinical studies
All manuscripts should comply with the ICMJE guidelines for publication of clinical research and a completed CONSORT checklist must be included with all submissions.

Clinical trial registration	This is no direct relevance to the study.
Study protocol	This is no direct relevance to the study.
Data collection	This is no direct relevance to the study.
Outcomes	This is no direct relevance to the study.

Dual use research of concern

Policy information about dual use research of concern

Hazards

Could the accidental, deliberate or reckless misuse of agents or technologies generated in the work, or the application of information presented in the manuscript, pose a threat to:

No	Yes	
☒	☐	Public health
☒	☐	National security
☒	☐	Crops and/or livestock
☒	☐	Ecosystems
☒	☐	Any other significant area

Experiments of concern

Does the work involve any of these experiments of concern:

No	Yes	
☒	☐	Demonstrate how to render a vaccine ineffective
☒	☐	Confer resistance to therapeutically useful antibiotics or antiviral agents
☒	☐	Enhance the virulence of a pathogen or render a nonpathogen virulent
☒	☐	Increase transmissibility of a pathogen
☒	☐	Alter the host range of a pathogen
☒	☐	Enable evasion of diagnostic/detection modalities
☒	☐	Enable the weaponization of a biological agent or toxin
☒	☐	Any other potentially harmful combination of experiments and agents

Plants

Seed stocks	This is no direct relevance to the study.
Novel plant genotypes	This is no direct relevance to the study.
Authentication	This is no direct relevance to the study.

ChIP-seq

Data deposition

☐ Confirm that both raw and final processed data have been deposited in a public database such as GEO.

☐ Confirm that you have deposited or provided access to graph files (e.g. BED files) for the called peaks.

Data access links<br>*May remain private before publication.*	This is no direct relevance to the study.
Files in database submission	This is no direct relevance to the study.
Genome browser session<br>(e.g. UCSC)	This is no direct relevance to the study.

Methodology

Replicates	This is no direct relevance to the study.
Sequencing depth	This is no direct relevance to the study.
Antibodies	This is no direct relevance to the study.
Peak calling parameters	This is no direct relevance to the study.
Data quality	This is no direct relevance to the study.
Software	This is no direct relevance to the study.

Flow Cytometry

Plots

Confirm that:

- ☐ The axis labels state the marker and fluorochrome used (e.g. CD4-FITC).
- ☐ The axis scales are clearly visible. Include numbers along axes only for bottom left plot of group (a 'group' is an analysis of identical markers).
- ☐ All plots are contour plots with outliers or pseudocolor plots.
- ☐ A numerical value for number of cells or percentage (with statistics) is provided.

Methodology

Sample preparation	not This is no direct relevance to the study.
Instrument	This is no direct relevance to the study.
Software	This is no direct relevance to the study.
Cell population abundance	This is no direct relevance to the study.
Gating strategy	This is no direct relevance to the study.

☐ Tick this box to confirm that a figure exemplifying the gating strategy is provided in the Supplementary Information.

Magnetic resonance imaging

Experimental design

Design type	This is no direct relevance to the study.
Design specifications	This is no direct relevance to the study.
Behavioral performance measures	This is no direct relevance to the study.

Acquisition

Imaging type(s)	This is no direct relevance to the study.
Field strength	This is no direct relevance to the study.
Sequence & imaging parameters	This is no direct relevance to the study.
Area of acquisition	This is no direct relevance to the study.

Diffusion MRI ☐ Used ☒ Not used

Preprocessing

Preprocessing software	This is no direct relevance to the study.
Normalization	This is no direct relevance to the study.
Normalization template	This is no direct relevance to the study.
Noise and artifact removal	This is no direct relevance to the study.
Volume censoring	This is no direct relevance to the study.

Statistical modeling & inference

Model type and settings	This is no direct relevance to the study.
Effect(s) tested	This is no direct relevance to the study.

Specify type of analysis: ☐ Whole brain ☐ ROI-based ☐ Both

Statistic type for inference

(See Eklund et al. 2016)

This is no direct relevance to the study.

Correction

This is no direct relevance to the study.

Models & analysis

n/a	Involved in the study
☒	☐ Functional and/or effective connectivity
☒	☐ Graph analysis
☒	☐ Multivariate modeling or predictive analysis
